# A taxonomic review of Korean species of the Atheta Thomson subgenus Microdota Mulsant & Rey, with descriptions of two new species (Coleoptera, Staphylinidae, Aleocharinae)

**DOI:** 10.3897/zookeys.502.9420

**Published:** 2015-05-04

**Authors:** Seung-Gyu Lee, Kee-Jeong Ahn

**Affiliations:** 1 Department of Biology, Chungnam National University, Daejeon 305-764, Republic of Korea

**Keywords:** Coleoptera, Staphylinidae, Aleocharinae, *Atheta*, *Microdota*, new species, Korea

## Abstract

A taxonomic review of the Atheta Thomson subgenus Microdota Mulsant & Rey in Korea is presented. The subgenus is represented in Korea by 15 species including two new species, Atheta (Microdota) jangtaesanensis Lee & Ahn, **sp. n.** and Atheta (Microdota) pasniki Lee & Ahn, **sp. n.** Four species [Atheta (Microdota) kawachiensis Cameron, Atheta (Microdota) muris Sawada, Atheta (Microdota) spiniventris Bernhauer, and Atheta (Microdota) spinula (Sawada)] are new to the Korean Peninsula and two [Atheta (Microdota) formicetorum Bernhauer and Atheta (Microdota) subcrenulata Bernhauer] to South Korea. Two other species [Atheta (Microdota) kobensis Cameron and Atheta (Microdota) scrobicollis (Kraatz)] previously recorded in North Korea had been identified incorrectly. A key, descriptions, habitus photographs and illustrations of the diagnostic features are provided. Species distributions and diversity in East Asia are discussed.

## Introduction

Mulsant and Rey (1873) proposed the genus name *Microdota* and described seven species. Since Ganglbauer (1895) first treated it as a subgenus of *Atheta*, Fenyes (1920), Yosii and Sawada (1976), Smetana (2004), and others followed his concept. Later, many additional species were described or transferred to the subgenus *Microdota* from other subgenera (for example, see Lynch Arribálzaga 1884; Peyerimhoff 1938; Scheerpeltz 1976). Although they are common in diverse microhabitats, nothing is known of their biology and immature stages.

The Atheta subgenus Microdota contains 215 species in the Palaearctic region. In East Asia, 40 species and 20 species were recorded in China and in Japan respectively (Smetana 2004). Paśnik (2001) reported 13 species including three new species from North Korea. We found that two species, Atheta (Microdota) kobensis Cameron and Atheta (Microdota) scrobicollis (Kraatz), were incorrect identifications of other Atheta (Microdota) species. Smetana (2004) transferred two other species, *Atheta mortuorum* (Thomson) and *Atheta nana*, treated in the subgenus *Microdota* by Paśnik (2001), to the subgenera *Pachyatheta* Munster and *Badura* Mulsant & Rey, respectively. None was recorded in South Korea.

In this study we recognize 15 Atheta (Microdota) species in Korea including two new species, Atheta (Microdota) jangtaesanensis Lee & Ahn, sp. n. and Atheta (Microdota) pasniki Lee & Ahn, sp. n. Four species, Atheta (Microdota) kawachiensis Cameron, Atheta (Microdota) muris Sawada, Atheta (Microdota) spiniventris Bernhauer, and Atheta (Microdota) spinula (Sawada) are newly added to the Korean fauna and two other species, Atheta (Microdota) formicetorum Bernhauer and Atheta (Microdota) subcrenulata Bernhauer, are identified for the first time in South Korea. A key to Korean Atheta (Microdota) species, descriptions, habitus photographs, and line drawings of diagnostic characters are provided.

The first author studied North Korean species in the Institute of Systematics and Evolution of Animals (ISEA), Kraków, Poland. All the other examined specimens are deposited in the Chungnam National University Insect Collection (CNUIC), Daejeon, Korea. Type specimens of Atheta (Microdota) species were also borrowed from the Field Museum of Natural History (FMNH), Chicago, USA, Museum für Naturkunde (MNHB), Berlin, Germany and the Natural History Museum (NHM), London, UK to have more reliable identifications. The explanation of labels is placed in square brackets in order to provide clearer collecting data in the material examined section.

## Results

### Genus *Atheta* Thomson, 1858

#### Subgenus Microdota

Taxon classificationAnimaliaColeopteraStaphylinidae

Mulsant & Rey, 1873

Microdota See Smetana (2004) for detailed synonymy.

##### Diagnosis.

Members of *Microdota* can be distinguished from other subgenera of *Atheta* by combination of the following characters: body small, parallel-sided; antennomere 2 distinctly longer than 3, 5–10 transverse; median region of prementum very narrow, without pseudopore; pronotum transverse, more than 1.2 times as wide as long, with midline of pubescence directed anteriorly in most; hypomera fully visible in lateral aspect; tarsal formula 4-5-5; flabellum reduced; abdominal tergites II–III without anterior macroseta in most, III–VI impressed in basal region, VI about as long as VII; internal sac of median lobe of aedeagus well developed (Fenyes 1920, Benick and Lohse 1974, Yosii and Sawada 1976, Seevers 1978).

##### Key to Korean species of the subgenus Microdota Mulsant & Rey

**Table:** 

1	Eyes small, shorter than tempora	**2**
–	Eyes medium or large, at least as long as or longer than tempora	**5**
2	Infraorbital carina complete; male abdominal sternite VIII with 8 macrosetae on each side of midline	**Atheta (Microdota) silvatica**
–	Infraorbital carina incomplete; male abdominal sternite VIII with 7 macrosetae on each side of midline	**3**
3	Abdominal tergites with transversely striate microsculpture	**Atheta (Microdota) hamgyongsani**
–	Abdominal tergites with imbricate microsculpture	**4**
4	Body smaller, less than 1.6 mm; antennomeres more transverse (Fig. 29); posterior margin of male abdominal tergite VIII slightly modified as in Fig. 38; male abdominal sternites V–VII with many small pores	**Atheta (Microdota) jangtaesanensis sp. n.**
–	Body larger, more than 1.6 mm; antennomeres less transverse (Fig. 33); posterior margin of male abdominal tergite VIII different as in Fig. 42; male abdominal sternites V–VII without many small pores	**Atheta (Microdota) pasniki sp. n.**
5	Infraorbital carina incomplete	**6**
–	Infraorbital carina complete	**8**
6	Eyes about as long as tempora; antennomeres more transverse, 11 slightly longer than preceding two combined (Fig. 28)	**Atheta (Microdota) palleola**
–	Eyes slightly longer than tempora; antennomeres less transverse, 11 as long as preceding two combined (Fig. 35)	**7**
7	Body reddish brown; abdominal tergites with transversely striate microsculpture	**Atheta (Microdota) sogamensis**
–	Body yellowish brown; abdominal tergites with imbricate microsculpture	**Atheta (Microdota) spinula**
8	Antennomere 11 slightly longer than preceding two combined (Figs 28, 34); posterior margin of male abdominal tergite VIII with process (Figs 37, 43)	**9**
–	Antennomere 11 as long as or shorter than preceding two combined (Figs 29–33, 35–36); posterior margin of male abdominal tergite VIII without processes (Figs 38–42, 44–45)	**10**
9	Labrum with about 11–12 macrosetae on each side of midline; mandibles with denticles in molar region; posterior margin of male abdominal tergite VIII with two processes (Fig. 37); male abdominal sternite VIII with 8 macrosetae on each side of midline	**Atheta (Microdota) formicetorum**
–	Labrum with about 8 macrosetae on each side of midline; mandibles without denticles in molar region; posterior margin of male abdominal tergite VIII with more than two processes (Fig. 43); male abdominal sternite VIII with 7 macrosetae on each side of midline	**Atheta (Microdota) spiniventris**
10	Abdominal tergites with imbricate microsculpture	**11**
–	Abdominal tergites with reticulate microsculpture (Sawada 1974: figs 10F, 11F, 18F)	**3**
11	Antennomere black; abdominal tergite VIII with 5 macrosetae on each side of midline (Paśnik 2001: fig. 27)	**Atheta (Microdota) kangsonica**
–	Antennomere yellowish brown to dark brown; abdominal tergite VIII with 4 macrosetae on each side of midline (Figs 37–45)	**12**
12	Body surface less glossy; pronotum, elytra and abdominal tergites II–IV reddish brown and darker; male sternite VIII with 7 macrosetae on each side of midline	**Atheta (Microdota) kawachiensis**
–	Body surface more glossy; pronotum, elytra and abdominal tergites II–IV yellowish brown and brighter; male sternite VIII with 8 macrosetae on each side of midline	**Atheta (Microdota) muris**
13	α-sensillum of epipharynx shorter (Sawada 1974: fig. 18B); abdominal tergites with slightly reticulate microsculpture; male abdominal sternites V–VII without many small pores	**Atheta (Microdota) koreana**
–	α-sensillum of epipharynx longer (Sawada 1974: figs 10B; 11B); abdominal tergites with distinctly reticulate microsculpture; male abdominal sternites V–VII with many small pores	**14**
14	Paramedian apophyses of internal sac longer, laterally produced basal plate narrower, copulatory piece less obtuse (Sawada 1974: fig. 10J); spermathecal duct less elongate (Sawada 1974: fig. 10N)	**Atheta (Microdota) amicula**
–	Paramedian apophyses of internal sac shorter, laterally produced basal plate broader, copulatory piece more obtuse (Sawada 1974: fig. 11J–K); spermathecal duct more elongate (Sawada 1974: fig. 11N)	**Atheta (Microdota) subcrenulata**

#### Atheta (Microdota) amicula

Taxon classificationAnimaliaColeopteraStaphylinidae

(Stephens, 1832)

Fig. 1

Aleochara amicula Stephens, 1832: 132.
Aleochara picipennis Stephens, 1832: 132 (as valid species); Fenyes 1920: 186; Smetana 2004: 384 (as synonym of *Atheta amicula*).
Homalota sericea Mulsant & Rey, 1852: 41 (as valid species); Fenyes 1920: 186; Brundin 1948: 32; Smetana 2004: 384 (as synonym of *Atheta amicula*).
Homalota subsericea Wollaston, 1864: 540 (as valid species); Fenyes 1920: 186; Smetana 2004: 384 (as synonym of *Atheta amicula*).
Homalota jezabel Saulcy, 1865: 438 (as valid species); Fenyes 1920: 186; Smetana 2004: 384 (as synonym of *Atheta amicula*).
Microdota terricola Mulsant & Rey, 1873b: 351 (as valid species); Fenyes 1920: 186; Smetana 2004: 384 (as synonym of *Atheta amicula*).
Homalota meludyi Quedenfeldt, 1884: 366 (as valid species); Fenyes 1920: 186; Smetana 2004: 384 (as synonym of *Atheta amicula*).
Atheta (Microdota) amicula attarum Bernhauer, 1929: 201 (as valid species); Smetana 2004: 384 (as synonym of *Atheta amicula*).
Atheta (Microdota) nuda G. Benick, 1975: 4 (as valid species); Smetana 2004: 384 (as synonym of *Atheta amicula*).
Atheta (Microdota) amicula : Brundin 1948: 32; Palm 1970: 190; G. Benick and Lohse 1974: 163; Sawada 1974: 164; Pace 1990: 906; Paśnik 2001: 207; Smetana 2004: 384 (as valid species).

##### Material examined.

Syntype, 3 exx., labeled as in Figs 82–83. NORTH KOREA: 3 exx., Corea sept 1987 Hyjćhon & vicin. Exped. ISEZ Cr. [North Korea, Jagang Prov., Huicheon-si, 1987, ISEA]; 3 exx., Corea sept. Kangvon–do 9–14 X 1991 [North Korea, Gangwon Prov., 9–14.x.1991]; 3 exx., Corea sept. 1987 Tanćhon & vic. Exped. ISEZ Cr.; 6 exx., Korea 5-8.6.1974 prov. Kesong-si Exp. Inst.Zool.Cr. [North Korea, Gyeonggi Prov. Gaeseong-si, 5–8.vi.l974, ISEA]; 4 exx., Korea 16-18.6.74. Kymgang-san Mts. Exp. Inst.Zool.Cr. [North Korea, Gangwon Prov., Mt. Geumgangsan, 16–18.vi.1974, ISEA].

##### Description.

**Figures 1–15.**
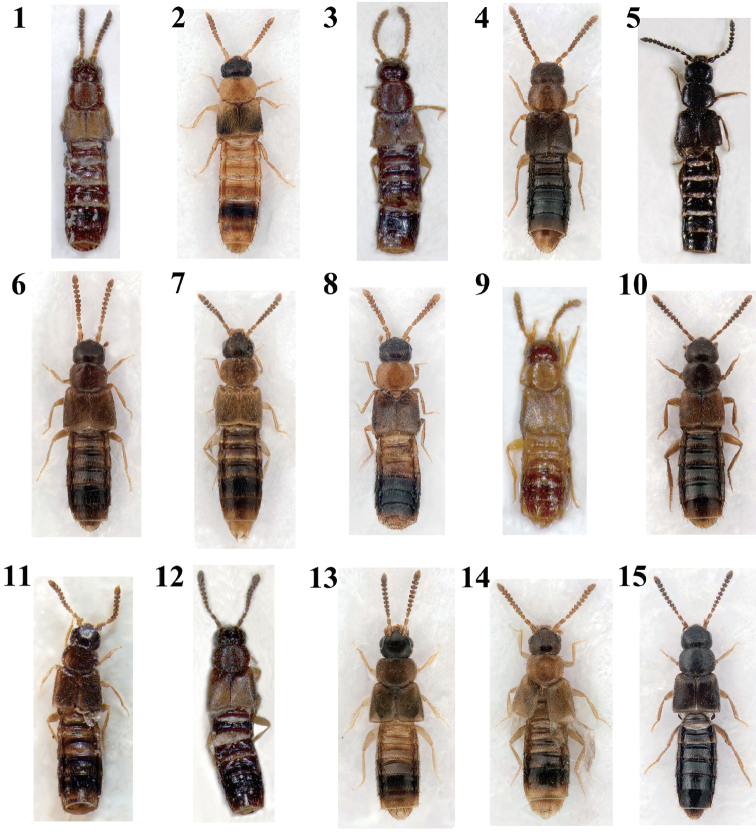
Habitus: **1** Atheta (Microdota) amicula, 1.8 mm **2** Atheta (Microdota) formicetorum, 2.4 mm **3** Atheta (Microdota) hamgyongsani, 1.7 mm **4** Atheta (Microdota) jangtaesanensis sp. n., 1.4 mm **5** Atheta (Microdota) kangsonica, 2.6 mm **6** Atheta (Microdota) kawachiensis, 1.8 mm **7** Atheta (Microdota) koreana, 1.6 mm **8** Atheta (Microdota) muris, 1.7 mm **9** Atheta (Microdota) palleola, 1.6 mm **10** Atheta (Microdota) pasniki sp. n., 2.1 mm **11** Atheta (Microdota) silvatica, 1.9 mm **12** Atheta (Microdota) sogamensis, 2.0 mm **13** Atheta (Microdota) spiniventris, 1.8 mm **14** Atheta (Microdota) spinula **15** Atheta (Microdota) subcrenulata, 2.0 mm.

Length 1.6–2.0 mm. Body (Fig. 1) slender and parallel-sided, more or less flattened; surface fairly glossy and densely pubescent, with fine microsculpture. Body usually reddish brown to dark brown; head and abdomen slightly darker than other parts; elytra slightly paler than pronotum; legs yellowish brown. *Head.* Quadrate, about as wide as long, widest across eyes, slightly narrower than pronotum; eyes moderate in size and slightly prominent, about 1.0–1.2 times longer than tempora; infraorbital carina complete; gular sutures moderately separated, diverged basally. Antennae dilated apically, slightly longer than head and pronotum combined; antennomeres 1–3 elongate, 1 longest, 4–10 distinctly transverse, 11 longer than wide, about as long as preceding two combined. *Thorax.* Pronotum transverse, approximately 1.3 times wider than long, widest at apical third; pubescence directed anteriorly in midline. Elytra slightly transverse, slightly wider than pronotum, elytron approximately 1.7 times longer than wide, pubescence directed posteriorly and postero-laterally; postero-lateral margin almost straight; hind wings fully developed. *Legs.* Slender and long, with dense pubescence and setae; tibiae with two spurs at apex; meso- and metatarsomeres 1–4 subequal in length. *Abdomen.* Parallel-sided, widest at middle; surface distinctly glossy and densely pubescent; male tergite VIII with 4 macrosetae on each side of midline, posterior margin slightly emarginate, with slight crenation; male sternite VIII with 7 macrosetae on each side of midline, posterior margin rounded; posterior margin of female tergite VIII subtruncate; posterior margin of female sternite VIII broadly rounded, with long and short marginal setae. *Genitalia.* Median lobe elongated oval; apical process convergent at apex in ventral aspect. Spermatheca with relatively large bursa; duct recurved apically.

##### Distribution.

Korea (North), China (Beijing), Cyprus, Israel, Europe (Austria, Azores, Croatia, Czech Republic, Denmark, Estonia, Faeroe Islands, Finland, France, Great Britain, Germany, Georgia, Greece, Hungary, Iceland, Ireland, Italy, Lithuania, Luxembourg, Malta, Macedonia, The Netherlands, Norway, Poland, Portugal, Slovakia, Spain, Sweden, Switzerland and Ukraine), Russia (North European Territory and West Siberia), Neotropical region and North Africa (Algeria, Canary Islands, Egypt, Morocco, Madeira Archipelago).

##### Remarks.

This species was recorded by Paśnik (2001) in North Korea and a dissected specimen was unavailable. Accordingly, we could not describe the mouthparts and aedeagus in detail. This species has been known to be often found on mushrooms (Palm 1970).

#### Atheta (Microdota) formicetorum

Taxon classificationAnimaliaColeopteraStaphylinidae

Bernhauer, 1907

Figs 2 , 28 , 37 , 46 , 55 , 64 , 73

Atheta (Microdota) formicetorum Bernhauer, 1907: 400; Paśnik 2001: 206; Smetana 2004: 385 (as valid species).
Atheta (Amidobia) formicetorum : Sawada 1974: 162 (as valid species).

##### Material examined.

Syntype, 1♀, labeled as in Fig. 84. NORTH KOREA: 1 ex., Corea sept. Kangvon–do 9–14 X 1991 [North Korea, Gangwon Prov., 9–14.x.1991]. SOUTH KOREA: Chungbuk Prov.: 3 exx., Danyang-gun, Yeongchun-myeon, Mt. Taehwasan, 14.vii–14.viii.2001, KJ Ahn, SJ Park, CW Shin, FIT;; Chungnam Prov.: 5 exx., Daejeon-si, Dong-gu, Daeseong-dong, Mt. Sikjangsan, Secheon park, 30.vii.2000, MH Kim, mushroom; 10 exx., Gongju-si, Banpo-myeon, Sangsin-ri, Mt. Gyeryongsan, 26.viii.2001, MH Kim, mushroom; Gangwon Prov.: 46 exx., Yangyang-gun, Seo-myeon, Mt. Seoraksan, Osaekyaksu, 16.viii.2000, MH Kim, mushroom; Gyeongbuk Prov.: 51 exx., Sangju-si, Hwanam-myeon, imgok-ri, Mt. Cheongtaesan, 10.ix.2000, MH Kim, mushroom; 11 exx., same data as the former except ‘27.vii.2001’; 3 exx., Uljin-gun, Onjeong-myeon, Mt. Baekamsan, Sinseon valley, 12.viii.1999, HJ Kim, mushroom; Gyeonggi Prov.: 3 exx., Yangju-gun, Jangheung-myeon, Songchu-ri, Mt. Bukhansan, 26.vii–31.viii.2001, KJ Ahn, SJ Park, CW Shin, FIT.

##### Description.

Length 1.8–2.5 mm. Body (Fig. 2) subparallel-sided, surface glossy, densely pubescent. Body usually reddish yellow to yellowish brown; head almost black; abdominal segment VI darker than other segments. *Head.* Slightly transverse, approximately 1.1–1.2 times wider than long, widest across eyes, slightly narrower than pronotum; eyes distinctly large and prominent, about 1.8–2.0 times longer than tempora; infraorbital carina complete; gular sutures moderately separated; cervical carina complete. Antennae (Fig. 28) long and slender, dilated apically; antennomeres 1–3 elongate, 1 longest, 4–10 distinctly transverse, 11 longer than wide, about as long as preceding two combined. *Mouthparts.* Labrum transverse, anterior margin emarginate; two lateral sensilla and about 11–12 macrosetae present on each side of midline; α-sensillum setaceous, about twice as long as ε-sensillum, β- and γ-sensilla reduced. Mandibles asymmetrical, pointed apically, about 1.6–1.7 times as long as basal width; many denticles present in molar region; right one with small internal tooth, anterior margin serrulate; prostheca developed. Lacinia of maxilla with seven spines in distal comb region, two isolated spines present, distal comb region and isolated spines narrowly separated; maxillary palpus elongate, with pubescence and long setae; palpomere 1 about 1.6–1.8 times longer than wide, 2 about 2.5–2.6 times longer than wide, 3 slightly longer than 2, about 2.6–2.8 times as long as wide, 4 digitiform, filamentous sensilla reaching to apical half. Labium with ligula narrowly long, divided into two lobes in basal half; two medial setae narrowly separated; two basal pores closed together; lateral pseudopores, one setal pore and two real pore present on each side of prementum; palpus elongate, with many setulae; palpomere 1 largest and dilated basally, about 1.5–1.7 times longer than wide, with γ-setula close b-setula, 2 shortest, about 1.4–1.6 times longer than wide, 3 slightly dilated apically, about as long as 1, about 3.0–3.5 times longer than wide. *Thorax.* Pronotum distinctly transverse, approximately 1.3–1.4 times wider than long, widest in apical third; pubescence directed anteriorly in midline. Metanotal scutum with one long seta and about 3–4 short setae on each side of midline; mesocoxal cavities narrowly separated, mesoventral process pointed at apex, as long as or slightly longer than isthmus and metaventral process combined; length ratio of mesoventral process, isthmus and metaventral process 16:5:10. Elytra slightly wider than pronotum; elytron approximately 1.5–1.6 times longer than wide, pubescence directed posteriorly and postero-laterally; postero-lateral margin almost straight; hind wings fully developed; flabellum composited about four setose lobes. *Legs.* Slender and long, with dense pubescence and setae; tibiae with two spurs at apex; length ratio of tarsomeres 22:24:25:65 (protarsus); 26:29:29:32:64 (mesotarsus); 35:33:31:30:69 (metatarsus); one empodial seta present, shorter than claw. *Abdomen.* Parallel-sided, widest at middle, surface fairly glossy and densely pubescent, with imbricate microsculpture; macrochaetal arrangement of tergites II–VI 01-02 (or 12)-12-12-13;; male tergite VII with small and round tubercle in postero-median region; male tergite VIII (Fig. 37) with 4 macrosetae on each side of midline, posterior margin with two outer processes and two inner processes, outer process narrower and longer than inner process; male sternites VI–VII with many pores in anterior margin, VIII with 8 macrosetae on each side of midline, posterior margin convex, crenate in median region; posterior margin of female tergite VIII emarginate; posterior margin of female sternite VIII slightly emarginate at middle, with conspicuous and short marginal setae; minute setae present in median region. *Genitalia.* Median lobe (Figs 46, 55) elongated oval; apical process convergent at apex in ventral aspect. Apical lobe of paramerites (Fig. 64) small and globular, with four setae, a- and b-setae longer than c- and d-setae, b-seta longest, c- and d-setae short, subequal in length. Spermatheca (Fig. 73) with bursa large and fusiform; umbilicus absent; duct short, round apically.

##### Distribution.

Korea (South, North) and Japan.

##### Remarks.

This species is identified for the first time in South Korea. Most specimens were found on mushrooms in forest.

#### Atheta (Microdota) hamgyongsani

Taxon classificationAnimaliaColeopteraStaphylinidae

Paśnik, 2001

Fig. 3

Atheta (Microdota) hamgyongsani Paśnik, 2001: 211; Smetana 2004: 385 (as valid species).

##### Material examined.

Holotype, ♂, labeled as follows: ‘Corea septentr. Hamgjong-pukdo 2-6 X 1991 ISEZ, HOLOTYPE Atheta (Microdota) hamgyongsani sp. n. det. G. Paśnik 2000’ [North Korea, Hamgyeongbuk Prov., 2–6.x.1991, ISEA].

##### Description.

Length 1.6–1.8 mm. Body (Fig. 3) slender and parallel-sided, more or less flattened; surface distinctly glossy and densely pubescent, with fine microsculpture. Body usually reddish brown; head and abdomen slightly darker than other parts; antennae and legs paler. *Head.* Subquadrate, about as wide as long, widest across eyes, slightly narrower than pronotum; eyes small and slightly prominent, about 0.6–0.7 times longer than tempora; infraorbital carina incomplete; gular sutures moderately separated, dilated apically. Antennae dilated apically; antennomeres 1–3 elongate, 1 longest, 4–10 distinctly transverse, 11 longer than wide, about as long as preceding two combined. *Thorax.* Pronotum transverse, approximately 1.3 times wider than long, widest at apical third; pubescence directed posteriorly in midline. Elytra slightly transverse, slightly wider than pronotum, elytron approximately 1.5 times longer than wide, pubescence directed posteriorly and postero-laterally; postero-lateral margin slightly sinuate; hind wings fully developed. *Legs.* Slender and long, with dense pubescence and setae; tibiae with two spurs at apex; meso- and metatarsomeres 1–4 subequal in length. *Abdomen.* Parallel-sided, widest at middle; surface distinctly glossy and densely pubescent; male tergite VIII with 4 macrosetae on each side of midline, posterior margin subtruncate; male sternite VIII with 7 macrosetae on each side of midline, posterior margin rounded. *Genitalia.* Median lobe (Paśnik 2001: Figs 30–31) oval; apical process abruptly convergent at apex in ventral aspect.

##### Distribution.

Korea (North).

##### Remarks.

This species was recorded by Paśnik (2001) in North Korea and a dissected specimen was unavailable. Accordingly, we could not describe the mouthparts and aedeagus in detail.

#### Atheta (Microdota) jangtaesanensis

Taxon classificationAnimaliaColeopteraStaphylinidae

Lee & Ahn sp. n.

http://zoobank.org/68BC54E8-FC59-4131-BFD0-282F07B5E50B

Figs 4 , 16–21 , 29 , 38 , 47 , 56 , 65 , 74

##### Material examined.

Holotype, labeled as follows: ‘KOREA: Chungnam prov., Daejeon-si, Seo-gu, Jangan-dong, Mt. Jangtaesan, N36°13'03.3", E127°20'36.2", 258 m, 28 III 2012, DH Lee, TK Kim, SG Lee, leaf litter; HOLOTYPE, Atheta (Microdota) jangtaesanensis Lee and Ahn, Desig. S.-G. Lee and K.-J. Ahn 2015.’ Deposited in CNUIC, Daejeon. Paratypes, 11 exx. (total); 4 exx., same data as holotype; 6 exx., same data as holotype except ‘5 IV 2013, SG Lee’.

##### Description.

**Figures 16–21.**
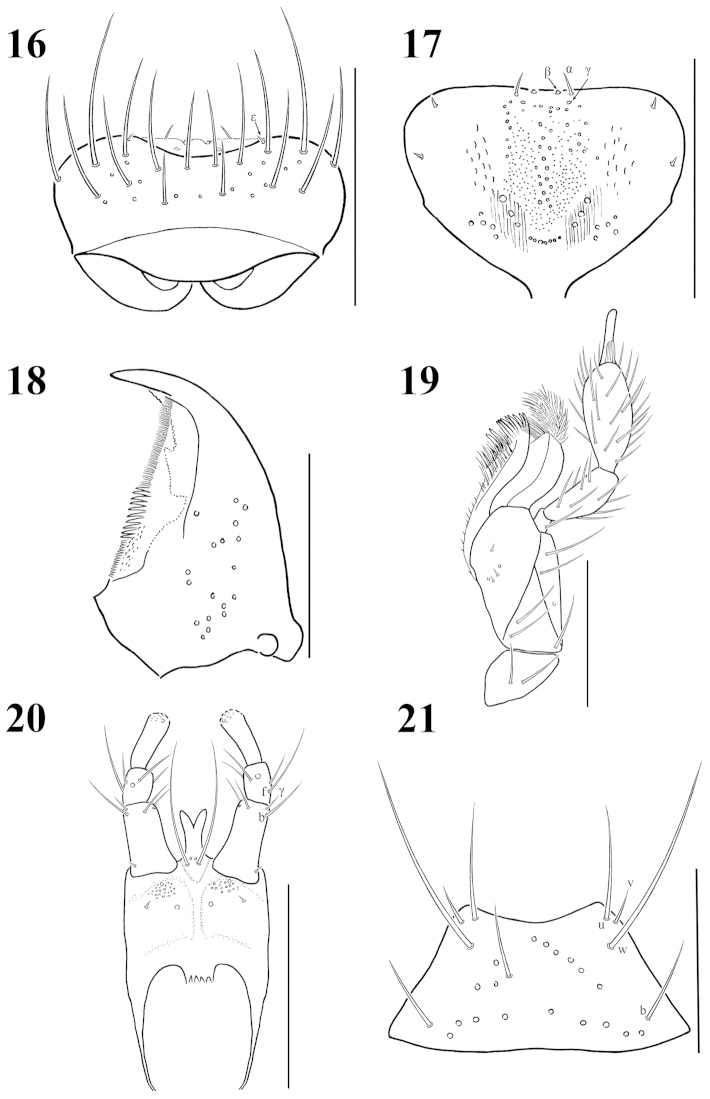
Mouthparts of Atheta (Microdota) jangtaesanensis sp. n.: **16** labrum, dorsal aspect **17** epipharynx, ventral aspect **18** right mandible, ventral aspect. **19** right maxilla, ventral aspect **20** labium, ventral aspect **21** mentum, ventral aspect. Scale bars: 0.1 mm.

Length 1.3–1.6 mm. Body (Fig. 4) slender and parallel-sided, more or less flattened dorso-ventrally; surface significantly glossy, densely pubescent, with fine microsculpture. Body usually reddish brown to reddish black; pronotum slightly paler than other parts; legs yellowish brown. *Head.* Subquadrate, approximately 1.0–1.1 times wider than long, widest behind eyes, slightly narrower than pronotum; eyes relatively small and prominent, about 0.7–0.8 times longer than tempora; gular sutures moderately separated, diverged basally; infraorbital carina incomplete; cervical carina almost complete. Antennae (Fig. 29) dilated apically; antennomeres 1–3 elongate, 1 longest, 4–10 transverse, 11 about as long as preceding two combined. *Mouthparts.* Labrum (Fig. 16) transverse, anterior margin emarginate; two lateral sensilla and about 8–9 macrosetae present on each side of midline; α-sensillum relatively long and setaceous, more than twice as long as ε-sensillum, β- and γ-sensilla reduced; epipharynx as in Fig. 17. Mandibles asymmetrical, pointed apically, approximately 1.7 times as long as basal width; right one (Fig. 18) with small internal tooth, anterior margin serrulate; prostheca developed. Lacinia of maxilla with seven spines in distal comb region, two isolated spines present; maxillary palpus elongate, with pubescence and long setae; palpomere 1 smallest and about twice as long as wide, 2 about 2.2–2.4 times longer than wide, 3 slightly longer than 2, about 2.6–2.8 times as long as wide, 4 digitiform, filamentous sensilla reaching to basal half (Fig. 19). Labium (Fig. 20) with ligula narrowly long, divided into two lobes in basal half; two medial setae narrowly separated; two basal pores closed together; lateral pseudopores, one setal pore and two real pores present on prementum; labial palpus elongate, with many setulae; palpomere 1 largest, about twice longer than wide, with γ-setula close b-setula, 2 shortest, about as long as wide, 3 dilated apically, about 2.4–2.5 times longer than wide. Mentum (Fig. 21) trapezoidal, anterior margin emarginate, v-seta relatively long. *Thorax.* Pronotum transverse, approximately 1.2–1.3 times wider than long, widest at apical third; pubescence directed anteriorly in midline. Metanotal scutum with one long seta and about 1–2 short setae on each side of midline; mesocoxal cavities narrowly separated, mesoventral process distinctly pointed at apex, about as long as isthmus and metaventral process combined; length ratio of mesoventral process, isthmus and metaventral process 13:9:4. Elytra slightly wider than pronotum; elytron approximately 1.7 times longer than wide, pubescence directed postero-laterally; postero-lateral margin almost straight; hind wings fully developed; flabellum composited about one setose lobe. *Legs.* Slender and long, with dense pubescence and setae; tibia with two spurs at apex; length ratio of tarsomeres 17:20:20:42 (protarsus); 20:25:24:23:44 (mesotarsus); 29:30:30:28:57 (metatarsus); one empodial seta present, shorter than claw. *Abdomen.* Parallel-sided, widest at middle; surface fairly glossy and densely pubescent, with fine and imbricate microsculpture; macrochaetal arrangement of tergites II–VI 01-02-12-12-13; male tergite VIII with 4 macrosetae on each side of midline, posterior margin truncate, slightly emarginate in median region; male sternites V–VII with many pores in anterior margin, VIII with 7 macrosetae on each side of midline, posterior margin round; posterior margin of female tergite VIII (Fig. 38) subtruncate; posterior margin of female sternite VIII broadly rounded, slightly emarginate at middle, with long and short marginal setae; minute setae present in median region. *Genitalia.* Median lobe (Figs 47, 56) entirely oval, apical process narrow apically, blunt at apex in ventral aspect. Apical lobe of paramerites (Fig. 65) elongate and subparallel-sided with four setae, b-seta longest, other setae subequal in length. Spermatheca (Fig. 74) with conical shaped umbilicus, duct sinuate and coiled apically.

**Figures 22–27.**
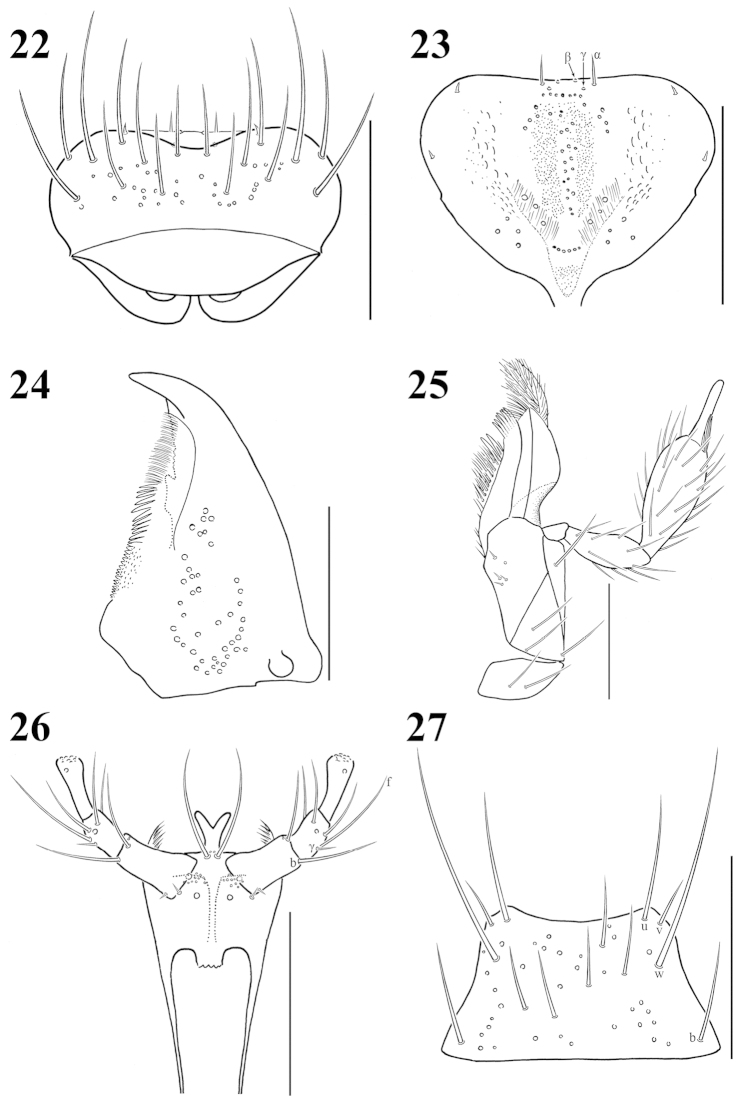
Mouthparts of Atheta (Microdota) pasniki sp. n.: **22** labrum, dorsal aspect **23** epipharynx, ventral aspect **24** right mandible, ventral aspect. **25** right maxilla, ventral aspect **26** labium, ventral aspect **27** mentum, ventral aspect. Scale bars: 0.1 mm.

**Figures 28–36.**
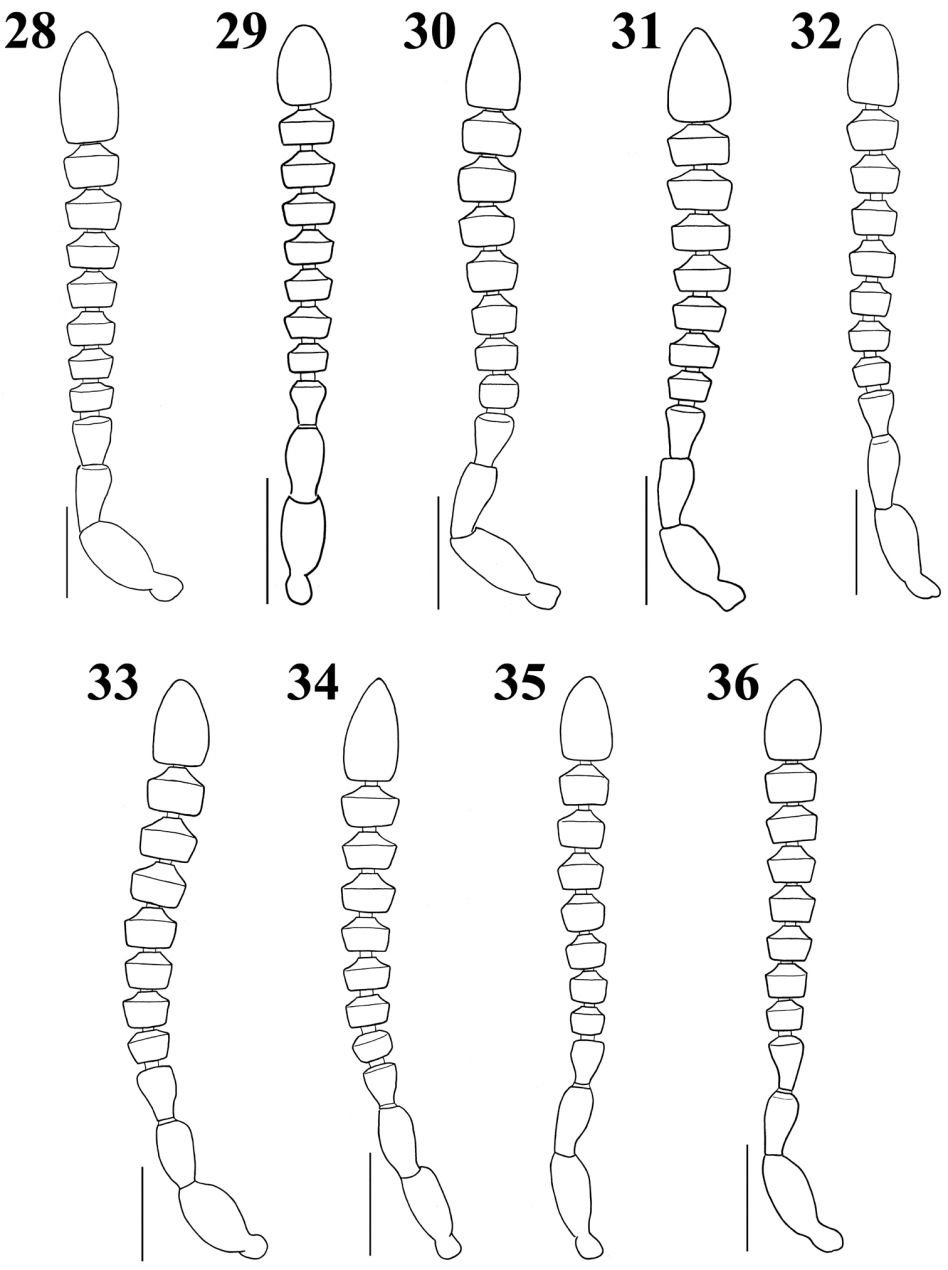
Antenna: **28** Atheta (Microdota) formicetorum **29** Atheta (Microdota) jangtaesanensis sp. n. **30** Atheta (Microdota) kawachiensis **31** Atheta (Microdota) koreana **32** Atheta (Microdota) muris **33** Atheta (Microdota) pasniki sp. n. **34** Atheta (Microdota) spiniventris **35** Atheta (Microdota) spinula **36** Atheta (Microdota) subcrenulata. Scale bars: 0.1 mm.

**Figures 37–45.**
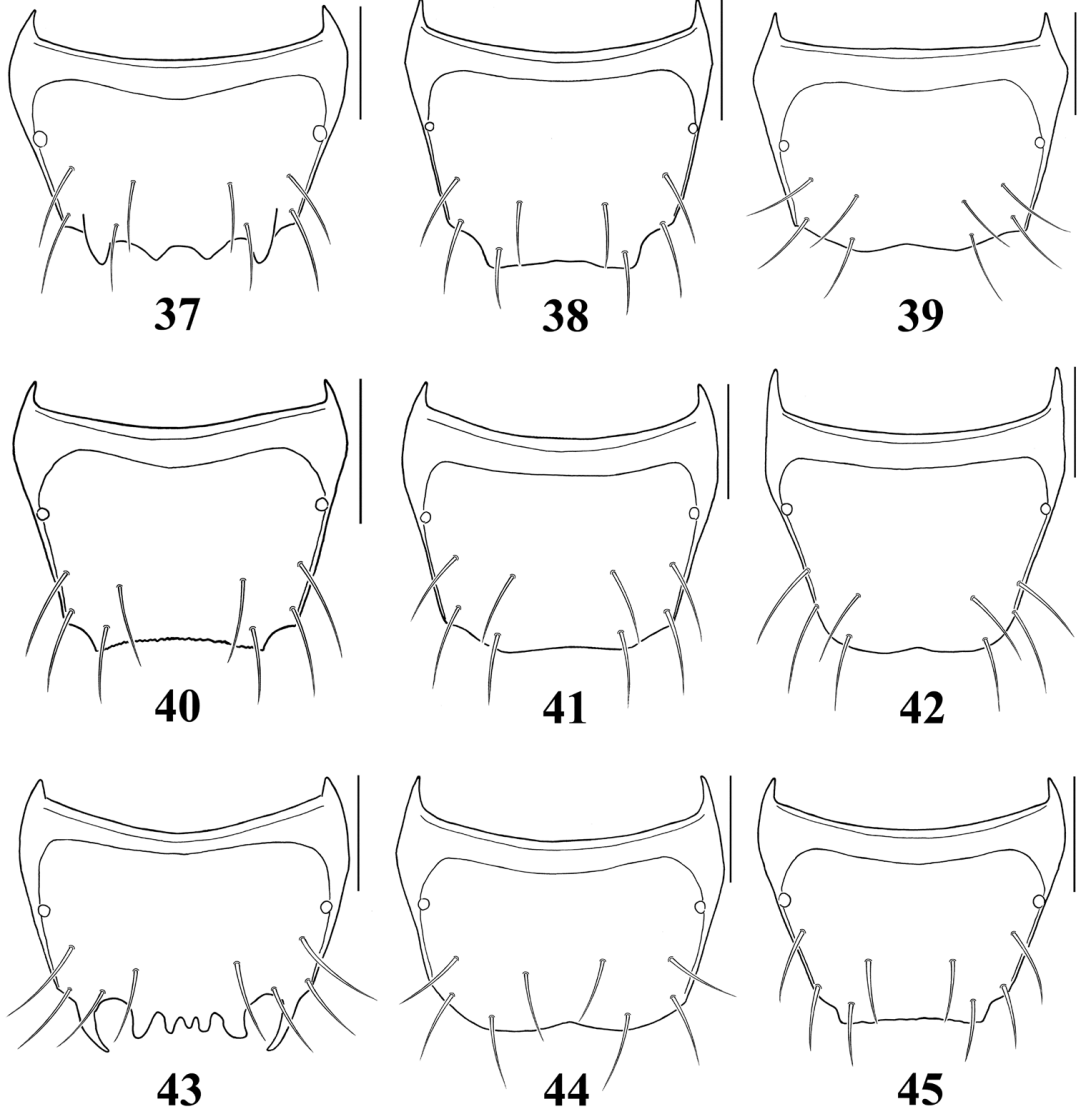
Male tergite VIII, dorsal aspect: **37** Atheta (Microdota) formicetorum **38** Atheta (Microdota) jangtaesanensis sp. n. **39** Atheta (Microdota) kawachiensis **40** Atheta (Microdota) koreana **41** Atheta (Microdota) muris **42** Atheta (Microdota) pasniki sp. n. **43** Atheta (Microdota) spiniventris **44** Atheta (Microdota) spinula **45** Atheta (Microdota) subcrenulata. Scale bars: 0.1 mm.

**Figures 46–54.**
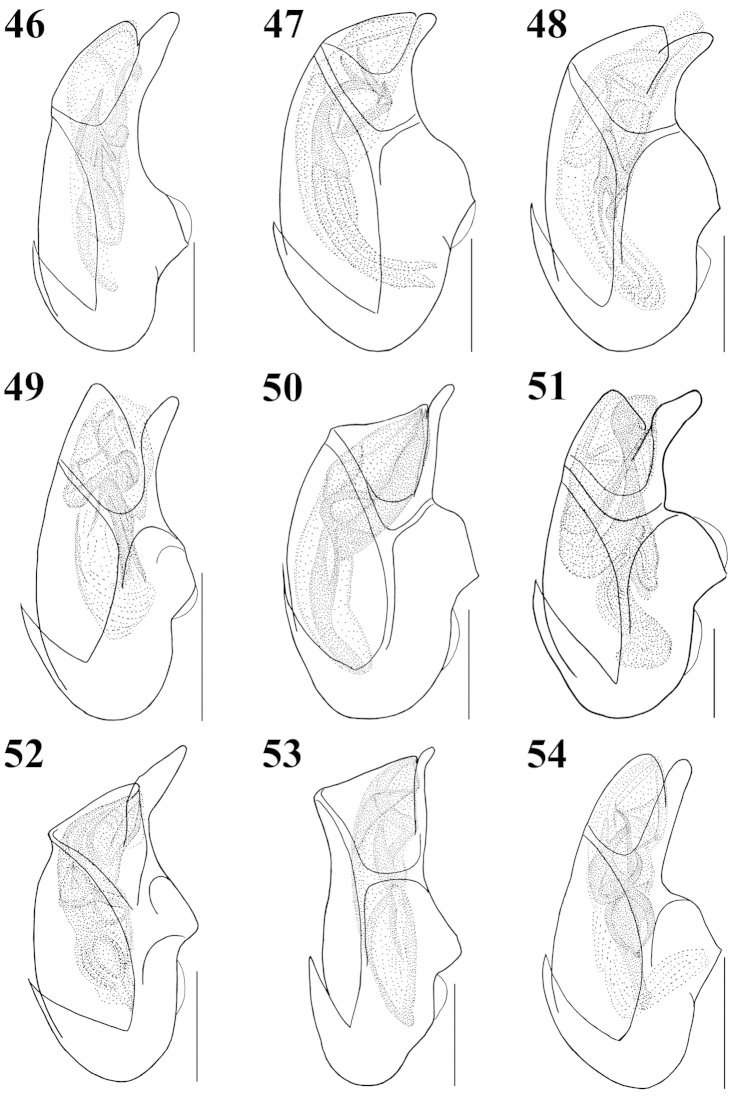
Median lobe of aedeagus, lateral aspect: **46** Atheta (Microdota) formicetorum **47** Atheta (Microdota) jangtaesanensis sp. n. **48** Atheta (Microdota) kawachiensis **49** Atheta (Microdota) koreana **50** Atheta (Microdota) muris **51** Atheta (Microdota) pasniki sp. n. **52** Atheta (Microdota) spiniventris **53** Atheta (Microdota) spinula **54** Atheta (Microdota) subcrenulata. Scale bars: 0.1 mm.

**Figures 55–63.**
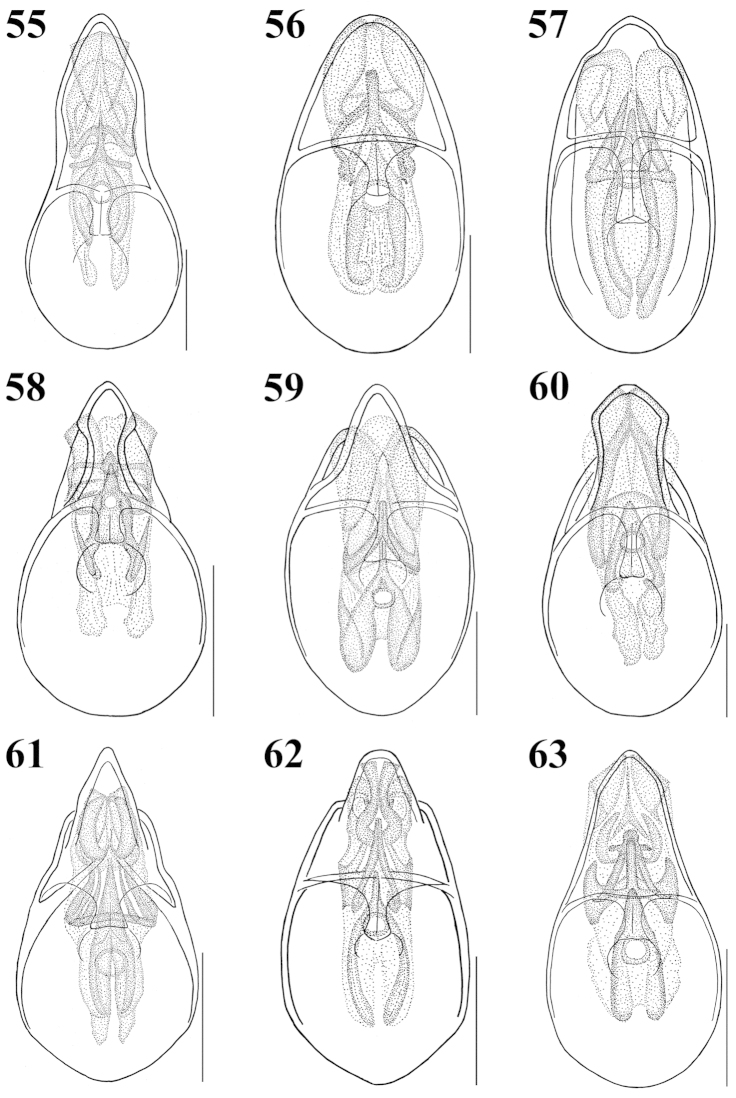
Median lobe of aedeagus, ventral aspect: **55** Atheta (Microdota) formicetorum **56** Atheta (Microdota) jangtaesanensis sp. n. **57** Atheta (Microdota) kawachiensis **58** Atheta (Microdota) koreana **59** Atheta (Microdota) muris **60** Atheta (Microdota) pasniki sp. n. **61** Atheta (Microdota) spiniventris **62** Atheta (Microdota) spinula **63** Atheta (Microdota) subcrenulata. Scale bars: 0.1 mm.

**Figures 64–72.**
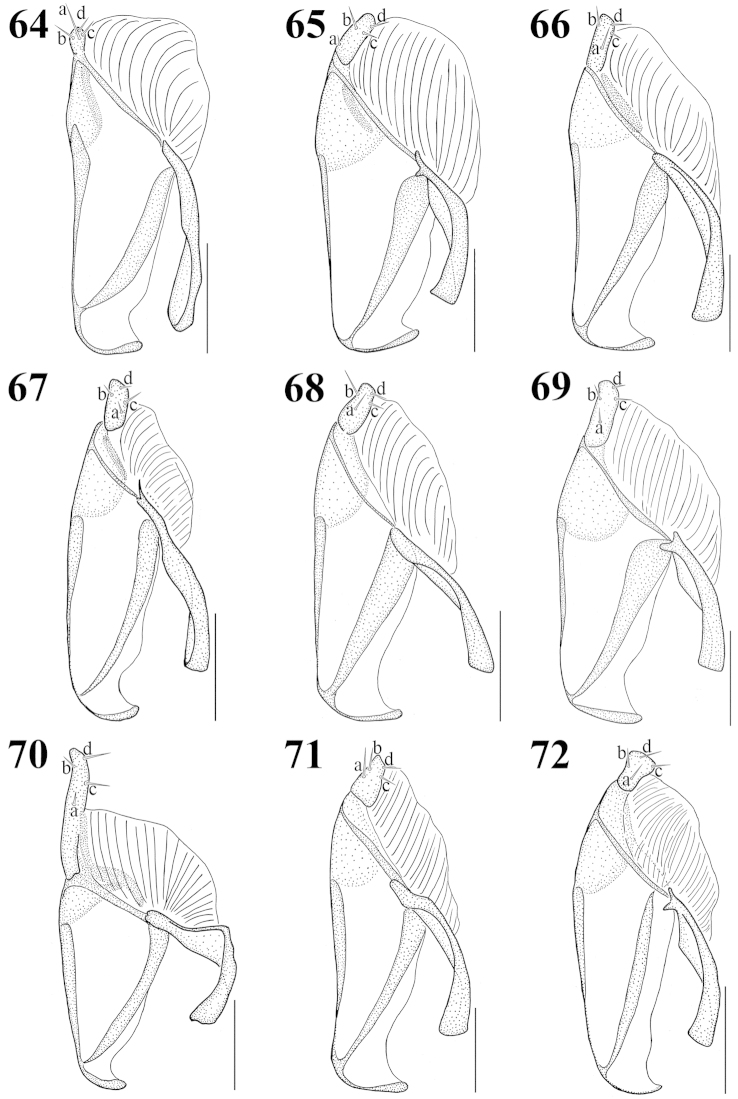
Paramere, lateral aspect: **64** Atheta (Microdota) formicetorum **65** Atheta (Microdota) jangtaesanensis sp. n. **66** Atheta (Microdota) kawachiensis **67** Atheta (Microdota) koreana **68** Atheta (Microdota) muris **69** Atheta (Microdota) pasniki sp. n. **70** Atheta (Microdota) spiniventris **71** Atheta (Microdota) spinula **72** Atheta (Microdota) subcrenulata. Scale bars: 0.1 mm.

**Figures 73–81.**
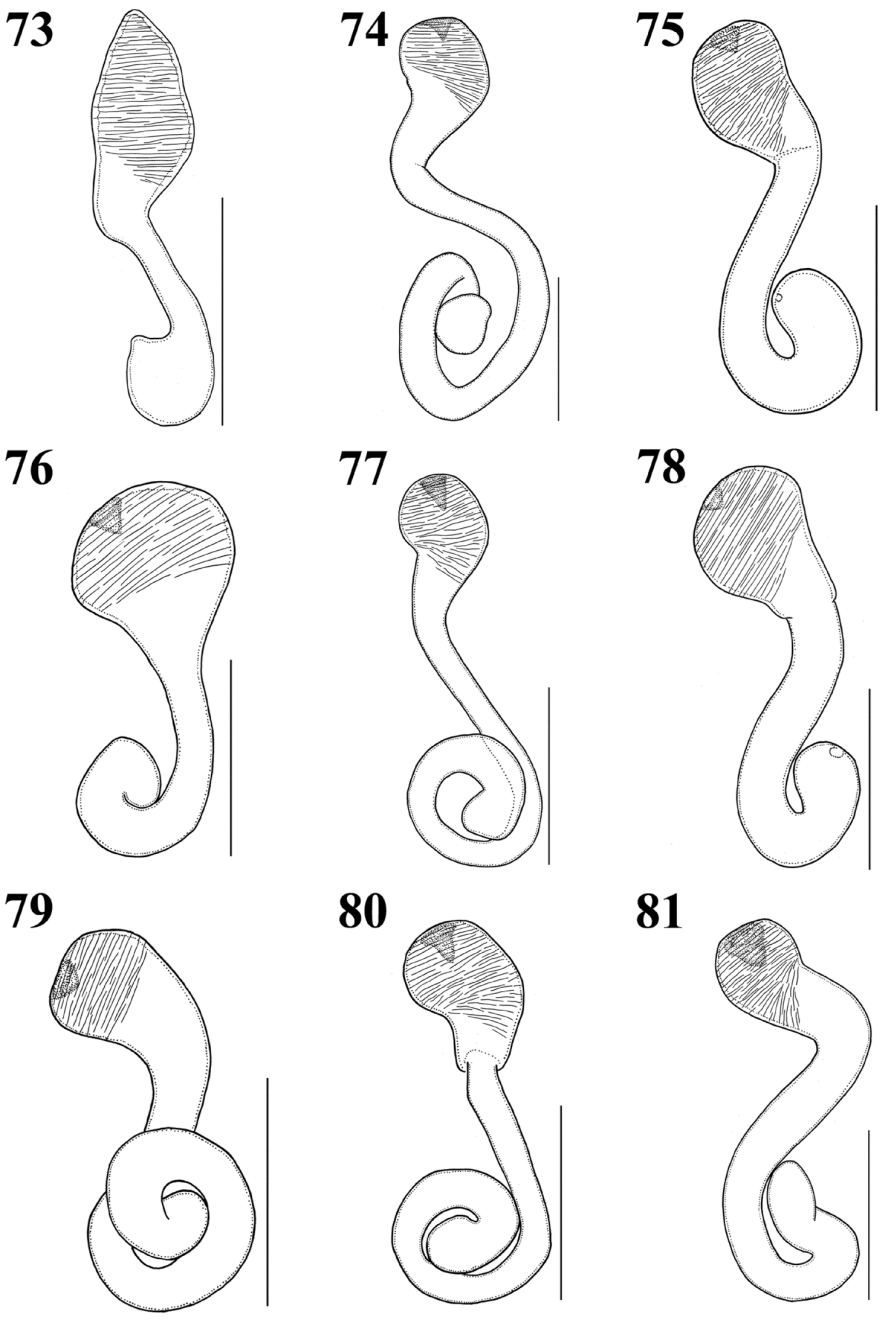
Spermatheca: **73** Atheta (Microdota) formicetorum **74** Atheta (Microdota) jangtaesanensis sp. n. **75** Atheta (Microdota) kawachiensis **76** Atheta (Microdota) koreana **77** Atheta (Microdota) muris **78** Atheta (Microdota) pasniki sp. n. **79** Atheta (Microdota) spiniventris **80** Atheta (Microdota) spinula **81** Atheta (Microdota) subcrenulata. Scale bars: 0.1 mm.

##### Distribution.

Korea (South).

##### Remarks.

This species is similar to Atheta (Microdota) muris, but can be distinguished by the small body; posterior margin of male tergite VIII with broad process; apical process of median lobe of aedeagus broad; annellus of internal sac relatively large; different shape of spermatheca. All specimens were collected by sifting leaf litter piled up in a ditch.

##### Etymology.

Named after the type locality Mt. Jangtaesan, where all of specimens were collected.

#### Atheta (Microdota) kangsonica

Taxon classificationAnimaliaColeopteraStaphylinidae

Paśnik, 2001

Fig. 5

Atheta (Microdota) kangsonica Paśnik, 2001: 209; Smetana 2004: 386 (as valid species).

##### Material examined.

Holotype, ♂, labeled as follows: ‘Korea Thesôsong, distr. Kangsô 8. 1971 1eg. Szeptycki, HOLOTYPE Atheta (Microdota) kangsonica sp. n. det. G. Paśnik 2000’ [North Korea, Pyeongannam Prov., Nampo-si, Gangseo-gun, Taeseongho, viii.1971, A. Szeptycki].

##### Description.

Length about 2.6 mm. Body (Fig. 5) parallel-sided, more or less flattened dorso-ventrally; surface glossy and densely pubescent, with fine microsculpture. Body usually dark brown; legs paler than other parts. *Head.* Slightly transverse, approximately 1.1 times wider than long, widest at middle, narrower than pronotum; eyes slightly large and prominent, about 1.1 times longer than tempora; infraorbital carina complete; gular sutures moderately separated, diverged basally. Antennae dilated apically; antennomeres 1–3 elongate, 1 longest, 4–7 slightly transverse, 8–10 distinctly transverse, 11 longer than wide, about as long as preceding two combined. *Thorax.* Pronotum slightly transverse, approximately 1.2–1.3 times wider than long, widest at apical third; pubescence directed anteriorly in midline. Elytra slightly transverse, slightly wider than pronotum, elytron approximately 1.7 times longer than wide, pubescence directed posteriorly and postero-laterally; postero-lateral margin almost straight; hind wings fully developed. *Legs.* Slender and long, with dense pubescence and setae; tibiae with two spurs at apex; meso- and metatarsomeres 1–4 subequal in length; one empodial seta present, shorter than claw. *Abdomen.* Parallel-sided, widest at middle; surface glossy and densely pubescent; male tergite VIII (Paśnik 2001: fig. 27) with 5 macrosetae on each side of midline, posterior margin emarginate; posterior margin of male sternite VIII convex and round. *Genitalia.* Median lobe (Paśnik 2001: figs 25–26) oval, apical process triangular, convergent at apex in ventral aspect.

##### Distribution.

Korea (North).

##### Remarks.

This species was recorded by Paśnik (2001) in North Korea and a dissected specimen was unavailable. Accordingly, we could not describe the mouthparts and aedeagus in detail.

#### Atheta (Microdota) kawachiensis

Taxon classificationAnimaliaColeopteraStaphylinidae

Sawada, 1974

Figs 6 , 30 , 39 , 48 , 57 , 66 , 75

Atheta (Amidobia) kawachiensis Sawada, 1974: 158.
Atheta (Microdota) kawachiensis : Smetana 2004: 386 (as valid species).

##### Material examined.

SOUTH KOREA: Gangwon Prov.: 32 exx., Sokcho-si, Mt. Seoraksan, Hwaamsa, 21.vi.2002, JS Park, sifting.

##### Description.

Length 1.6–2.0 mm. Body (Fig. 6) slender and parallel-sided, more or less flattened dorso-ventrally; surface glossy, densely pubescent, with fine microsculpture. Body usually reddish brown to dark brown; head and abdomen dark brown to black; pronotum slightly darker than elytra; legs yellowish brown. *Head.* Subquadrate, approximately 1.0–1.1 times wider than long, widest across eyes, slightly narrower than pronotum; eyes moderate in size and prominent, about 1.0–1.2 times longer than tempora; gular sutures moderately separated, diverged basally; infraorbital carina complete; cervical carina complete. Antennae (Fig. 30) dilated apically; antennomeres 1–3 elongate, 1 longest, 4–10 slightly transverse to transverse, 11 longer than wide, about as long as preceding two combined. *Mouthparts.* Labrum transverse, anterior margin emarginate; two lateral sensilla and about 8 macrosetae present on each side of midline, α-sensillum setaceous, slightly longer than ε-sensillum, β- and γ-sensilla reduced. Mandibles asymmetrical, pointed apically, about 1.6–1.7 times as long as basal width; anterior margin serrulate; right one with small internal tooth; prostheca developed. Lacinia of maxilla with seven spines in distal comb, two isolated spines present; maxillary palpus elongate, with pubescence and long setae; palpomere 1 smallest and about 1.6–1.8 times as long as wide, 2 about 2.5–2.7 times longer than wide, 3 longer than 2, about 2.4–2.5 times as long as wide, 4 digitiform, filamentous sensilla reaching to basal half. Labium with ligula divided into two lobes in basal half; two medial setae narrowly separated; two basal pores contiguous; lateral pseudopores, one setal pore and two real pores present on prementum; labial palpus elongate, with many setulae; palpomere 1 largest and dilated basally, about 1.3–1.5 times longer than wide, with γ-setula close b-setula, 2 shortest, about 1.2–1.4 times longer than wide, 3 long and slender, about as long as 1, about 3.5–4.0 times longer than wide. *Thorax.* Pronotum transverse, approximately 1.2 times wider than long, widest at apical third; pubescence directed anteriorly in midline. Metanotal scutum with one long seta and about 1–2 short setae on each side of midline; mesocoxal cavities narrowly separated, mesoventral process pointed at apex, about twice longer than metaventral process. Elytra slightly wider than pronotum; elytron approximately 1.7–1.8 times longer than wide, pubescence directed postero-laterally; postero-lateral margin almost straight; hind wings fully developed; flabellum composited one long setose lobe. Legs. Slender and long, with dense pubescence and setae; tibia with two spurs at apex; length ratio of tarsomeres 16:17:19:49 (protarsus); 20:22:23:23:48 (mesotarsus); 28:28:28:27:60 (metatarsus); one empodial seta present, shorter than claw. *Abdomen.* Parallel-sided, widest at middle; surface distinctly glossy and densely pubescent, with fine and imbricate microsculpture; macrochaetal arrangement of tergites II–VI 01-02-12-12-13; male tergite VIII (Fig. 39) with 4 macrosetae on each side of midline, posterior margin slightly emarginate; male sternites V–VII with many pores in anterior margin, VIII with 7 macrosetae on each side of midline, posterior margin convex, slightly rounded, with long marginal setae; posterior margin of female tergite VIII similar to male; posterior margin of female sternite VIII slightly emarginate at middle, with long and short marginal setae; minute setae present in median region. *Genitalia.* Median lobe (Figs 48, 57) oval; apical process triangular, convergent at apex in ventral aspect. Apical lobe of paramerites (Fig. 66) subparallel-sided and elongate with four short setae; a-seta longest, slightly longer than other setae. Spermatheca (Fig. 75) with small umbilicus, duct one coiled apically.

##### Distribution.

Korea (South) and Japan.

##### Remarks.

All specimens were collected by sifting leaf litter in Gangwon province.

#### Atheta (Microdota) koreana

Taxon classificationAnimaliaColeopteraStaphylinidae

Bernhauer, 1923

Figs 7 , 31 , 40 , 49 , 58 , 67 , 76

Atheta (Microdota) koreana Bernhauer, 1923: 128; Cho and Ahn 2001: 32; Paśnik 2001: 208; Smetana 2004: 386 (as valid species).
Atheta (Amidobia) bulbosa Sawada, 1974: 179 (as valid species); Smetana 2004: 386 (as synonym of *Atheta koreana*).
Ischnopoda (Atheta) koreana : Yuh et al. 1985: 251 (as valid species).
Atheta koreana : Kim et al. 1994: 144 (as valid species).

##### Material examined.

Syntype, 1♀, labeled as in Fig. 85. NORTH KOREA: 1 ex., Korea 25.5.1974 Jonghen ad Dżuyr Exp. Inst.Zool.Cr. [North Korea, Hamgyeongbuk Prov., Gyeongseong-gun, Yonghyeon-ri, 25 v 1974, ISEA]. SOUTH KOREA: Chungbuk Prov.: 3 exx., Boeun-gun, Maro-myeon, Imgok-ri, Mt. Cheontaesan, 6.viii.2000, MH Kim, mushroom; Chungnam Prov.: 7 exx., Gongju-si, Mt. Gyeryongsan, N36°21'17.4", E127°14'55.7",, 1–18.vi.2004, KJ Ahn, SM Choi, JS Park, FIT; 1 ex., Gongju-si, Sangsin-ri, Mt. Gyeryongsan, N36°22'03.2", E127°12'50",, 31.v–18.vi.2004, SM Choi, JS Park, FIT; Gongju-si, Mt. Gyeryongsan, Eunseon-waterfall, N36°20'58.7", E127°12'41.3",, 1–18.vi.2004, SM Choi, JS Park, FIT; 6 exx., Gongju-si, Mt. Gyeryongsan, Donghaksa, N36°21'17.4", E127°14'55.7",, 1–18.vi.2004, SM Choi, JS Park, FIT; 9 exx., Gongju-si, Mt. Gyeryongsan, Dongwol, N36°19'39", E127°15'46.7",, KJ Ahn, SM Choi, JS Park, FIT; 5 exx., Gongju-si, Mt. Gyeryongsan, Gapsa, N36°22'03.2", E127°12'50",, 31.v–18.vi.2004, KJ Ahn, SM Choi, JS Park, FIT; Gongju-si, Mt. Gyeryongsan, Nammaetap, N36°21'11.8", E127°13'20.8",, SM Choi, JS Park, FIT; Gangwon Prov.: 3 exx., Hoengseong-gun, Gangrim-myeon, Bugok-ri, Mt. Chiaksan, 15.viii.2000, MH Kim, rotten mushroom (Boletaceae); 6 exx., Hongcheon-gun, Nae-myeon, Gyebangsan, Unduryeong, 18.viii.2000, MH Kim, rotten mushroom (Boletaceae); 4 exx., Hongcheon-gun, Naecheon-myeon, Garyeong fall, 25.v–20.vi.2002, KJ Ahn, SJ Park, JS Park, FIT; 9 exx., Pyeongchang-gun, Bangrim-myeon, Ungyo 2-ri, Mt. Baedeoksan, 12.vii.–16.viii.2001, KJ Ahn, SJ Park, CW Shin, FIT; 4 exx., Pyeongchang-gun, Jinbu-myeon, Mt. Odaesan, Sangwonsa, 18.v–23.vi.2002, SJ Park, FIT; 2 exx., Sokcho-si, Mt. Seoraksan, Biseondae, 30.vii–15.ix.2002, SJ Park, JS Park, FIT; 6 exx., Yeongwol-gun Yeongwol-eup, Taehawasan 14.viii.2001, MH Kim, rotten mushroom (Boletaceae); Gyeonggi Prov.: 3 exx., Gapyeong-gun, Buk-myeon, Mt. Myeongjisan, 25.vii.–30.viii.2001, KJ Ahn, SJ Park, CW Shin, FIT; 3 exx., Yangju-gun, Jangheung-myeon, Songchu-ri, Mt. Bukhansan, 24.iii.1998, YS Kim.

**Figures 82–91.**
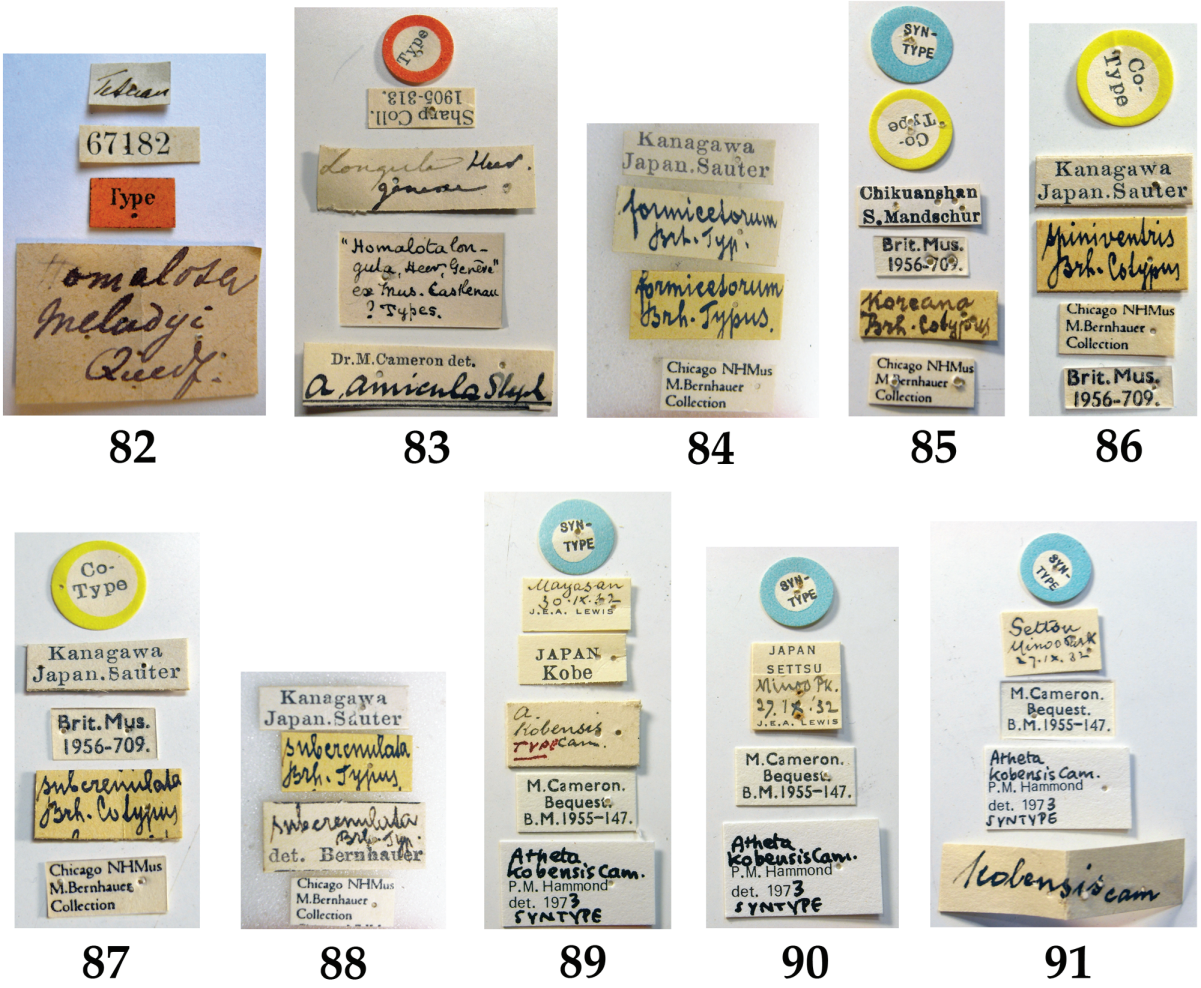
Label data of the type series: **82** Atheta (Microdota) amicula, syntype from NHM **83** Atheta (Microdota) amicula, syntype from MNHUB **84** Atheta (Microdota) formicetorum, syntype from FMNH **85** Atheta (Microdota) koreana, syntype from NHM **86** Atheta (Microdota) spiniventris, syntype from NHM **87** Atheta (Microdota) subcrenulata, syntype from NHM **88** Atheta (Microdota) subcrenulata, syntype from FMNH **89–91** Atheta (Microdota) kobensis, syntypes from NHM.

##### Description.

Length 1.5–1.8 mm. Body (Fig. 7) parallel-sided; surface glossy, densely pubescent, with fine microsculpture. Body usually yellowish brown to reddish brown; head and abdomen darker than other parts. *Head.* Slightly transverse, approximately 1.1–1.2 times wider than long, widest across eyes, slightly narrower than pronotum; eyes prominent, about 1.2–1.3 times longer than tempora; gular sutures moderately separated, diverged basally; infraorbital carina complete, cervical carina complete. Antennae (Fig. 31) dilated apically; antennomeres 1–3 elongate, 1 about as long as 11, 4–10 distinctly transverse, 11 longest, about as long as preceding two combined. *Mouthparts.* Labrum transverse, anterior margin emarginate, two lateral sensilla and about 8 macrosetae present on each side of midline; α-sensillum setaceous, more than twice as long as ε-sensillum, β- and γ-sensilla reduced. Mandibles asymmetrical, pointed apically, about 1.5–1.6 times as long as basal width; right one with small internal tooth, anterior margin serrulate; prostheca developed. Lacinia of maxilla with seven spines in distal comb, two isolated spines present; maxillary palpus elongate, with pubescence and long setae; palpomere 1 smallest and about 1.5–1.7 times as long as wide, 2 about 2.6–2.7 times longer than wide, 3 longer than 2, about 2.3–2.5 times as long as wide, 4 digitiform, filamentous sensilla reaching to basal half. Labium with ligula divided into two lobes in basal half; two medial setae narrowly separated; two basal pores close; lateral pseudopores, one setal pore and two real pores present on prementum; labial palpus elongate, with many setulae; palpomere 1 largest and dilated basally, about as long as wide, with γ-setula close b-setula, 2 shortest, about 1.4–1.6 times longer than wide, 3 slightly dilated apically, slightly shorter than 1, about 2.5 times longer than wide. *Thorax.* Pronotum transverse, approximately 1.2–1.3 times wider than long, widest at middle; pubescence directed anteriorly in midline. Metanotal scutum with one long seta and about 2–3 short setae on each side of midline; mesocoxal cavities narrowly separated, mesoventral process pointed at apex, longer than isthmus and metaventral process combined; length ratio of mesoventral process, isthmus and metaventral process 14:7:5. Elytra subquadrate and slightly dilated apically, slightly wider than pronotum; elytron approximately 1.6–1.7 times longer than wide, pubescence directed postero-laterally; postero-lateral margin almost straight; hind wings fully developed; flabellum composited about one setose lobe. *Legs.* Slender and long, with dense pubescence and setae; tibia with two spurs at apex; length ratio of tarsomeres 14:15:16:32 (protarsus); 17:19:20:20:40 (mesotarsus); 24:24:24:24:49 (metatarsus); one empodial seta present, shorter than claw. *Abdomen.* Parallel-sided, widest at middle; surface fairly glossy and densely pubescent, with slightly reticulate microsculpture; macrochaetal arrangement of tergites II–VI 01-02-12-12-13; male tergite VIII (Fig. 40) with 4 macrosetae on each side of midline, posterior margin truncate, with minute crenation; male sternite VIII with 7 macrosetae on each side of midline, posterior margin rounded; posterior margin of female tergite VIII subtruncate; posterior margin of female sternite VIII broadly rounded, slightly emarginate at middle, with long and short marginal setae; minute setae present in median region. *Genitalia.* Median lobe (Figs 49, 58) oval, apical process constricted in median region, convergent at apex in ventral aspect; internal sac complicated. Apical lobe of paramerites (Fig. 67) subparallel-sided and elongate, with four setae subequal in length. Spermatheca (Fig. 76) with very large bursa, duct one coiled apically.

##### Distribution.

Korea (South, North), China (Liaoning) and Japan.

##### Remarks.

This species is distinguished from similar species by the characters provided in Sawada (1974). Some specimens were found on mushrooms in forest areas.

#### Atheta (Microdota) muris

Taxon classificationAnimaliaColeopteraStaphylinidae

Sawada, 1974

Figs 8 , 32 , 41 , 50 , 59 , 68 , 77

Atheta (Amidobia) muris Sawada, 1974: 176.
Atheta (Microdota) muris : Smetana 2004: 387 (as valid species).

##### Material examined.

SOUTH KOREA: Chungnam Prov.: 1 ex., Buyeo-gun, Oesan-myeon, Gaedeok-ri, Mt. Wolmyeongsan, 1.vi.2000, US Hwang, HJ Kim, sifting; 26 exx., Daejeon-si, Chungnam National Univ., 13.v.2002, JS Park, sifting; 7 exx., Daejeon-si, Yuseong-gu, Chungnam National Univ., 26.ix.2002, SM Choi, JH Choi, sifting; 1 ex., Daejeon-si, Mt. Sikjangsan, Secheon park, 17.vii.2000, HJ Kim, mushroom; 1 ex., Gongju-si, Mt. Gyeryongsan, 23.vi.2000, HJ Kim, near stream; 1 ex., Hongseong-gun, Gwangcheon-eup, Oseosan, 20.vi.1999, HJ Kim, near stream; Gangwon Prov.: 6 exx., Pyeongchang-gun, Odaesan, Jeokmyeolbogung, 8.vii.1998, KJ Ahn, mushroom; Gyeonggi prov.: 6 exx., Namyangju-si, Sudong-myeon, Oebang-ri, Mt. Chukryeongsan, 13.ix.1999, US Hwang, HJ Kim, sifting.

##### Description.

Length 1.4–1.9 mm. Body (Fig. 8) slender and parallel-sided, more or less flattened dorso-ventrally; surface distinctly glossy, densely pubescent, with fine microsculpture. Body usually yellowish brown; head and abdominal segments V–VII dark brown to black; pronotum slightly paler than elytra. *Head.* Subquadrate, approximately 1.0–1.1 times wider than long, widest across eyes, slightly narrower than pronotum; eyes moderate in size and prominent, about 1.0–1.2 times longer than tempora; gular sutures moderately separated, diverged basally; infraorbital carina complete; cervical carina complete. Antennae (Fig. 32) dilated apically; antennomeres 1–3 elongate, 1 longest, 4–10 transverse, 11 longer than wide, slightly shorter than preceding two combined. *Mouthparts.* Labrum transverse, anterior margin emarginate; two lateral sensilla and about 8 macrosetae present on each side of midline; α-sensillum setaceous, slightly longer than ε-sensillum, β- and γ-sensilla reduced. Mandibles asymmetrical, pointed apically, about 1.5–1.6 times as long as basal width; right one with small internal tooth, anterior margin serrulate; prostheca developed. Lacinia of maxilla with seven spines in distal comb, two isolated spines present; maxillary palpus elongate, with pubescence and long setae; palpomere 1 smallest and about 1.8–2.0 times as long as wide, 2 about 2.5–2.7 times longer than wide, 3 slightly longer than 2, about 2.2–2.5 times as long as wide, 4 digitiform, filamentous sensilla reaching to basal half. Labium with ligula divided into two lobes in basal half; two medial setae narrowly separated; two basal pores close; lateral pseudopores and two real pores present on prementum; labial palpus elongate, with many setulae; palpomere 1 largest and dilated basally, about 1.5–1.7 times longer than wide, with γ-setula close b-setula, 2 shortest, about 1.4–1.6 times longer than wide, 3 about as long as 1, about 3.0 times longer than wide. *Thorax.* Pronotum transverse, approximately 1.3 times wider than long, widest at apical third; pubescence directed anteriorly in midline. Metanotal scutum with one long seta and about two short setae on each side of midline; mesocoxal cavities narrowly separated, mesoventral process pointed at apex, longer metaventral process; length ratio of mesoventral process, isthmus and metaventral process 15:8:6. Elytra slightly wider than pronotum; elytron approximately 1.5–1.6 times longer than wide, pubescence directed postero-laterally; postero-lateral margin almost straight; hind wings fully developed; flabellum composited one setose lobe. *Legs.* Slender and long, with dense pubescence and setae; tibia with two spurs at apex; length ratio of tarsomeres 18:20:20:44 (protarsus); 20:23:24:24:45 (mesotarsus); 31:31:31:30:53 (metatarsus); one empodial seta present, shorter than claw. *Abdomen.* Parallel-sided, widest at middle; surface distinctly glossy and densely pubescent, with fine and imbricate microsculpture; macrochaetal arrangement of tergites II–VI 01-02-12-12-13; male tergite VIII (Fig. 41) with 4 macrosetae on each side of midline, posterior margin truncate; male sternites V–VII with many pores in anterior margin, VIII with 8 macrosetae on each side of midline, posterior margin broadly rounded, with long marginal setae; posterior margin of female tergite VIII subtruncate; posterior margin of female sternite VIII slightly emarginate at middle, long and short marginal setae; minute setae present in median region. *Genitalia.* Median lobe (Figs 50, 59) oval, apical process abruptly convergent at apex in ventral aspect. Apical lobe of paramerites (Fig. 68) elongate and subparallel-sided with four setae; a- and b-setae longer than c- and d- setae. Spermatheca (Fig. 77) with duct relatively long, coiled apically.

##### Distribution.

Korea (South) and Japan.

##### Remarks.

Some specimens were found on mushrooms in forests.

#### Atheta (Microdota) palleola

Taxon classificationAnimaliaColeopteraStaphylinidae

(Erichson, 1837)

Fig. 9

Homalota palleola Erichson, 1837: 333.
Atheta (Microdota) palleola : Palm 1970: 186; Paśnik 2001: 209; Smetana 2004: 387 (as valid species).

##### Material examined.

Syntype, 4 exx., labeled as follows: ‘5451, pallola Er. Berol. Weis.’. NORTH KOREA: 2 exx., Korea, Jongak-san Pnjongjang-si, lg. Pawlowski, 8.1971 [North Korea, Pyeongannam Prov. Pyeongyang-si, Mt. Yongaksan, viii.1971, J. Pawlowski]; 4 exx., Korea 1-3.6.1974 Sujang-san Mt. Exp. Inst.Zool.Cr. [North Korea, Hwanghae Prov. Mt. Suyangsan, 1–3.vi.1974, ISEA].

##### Description.

Length 1.4–1.8 mm. Body (Fig. 9) slender and parallel-sided; surface glossy and densely pubescent, with fine microsculpture. Body usually yellowish brown; head and abdominal tergites VI–VII darker than other parts. *Head.* Slightly transverse, approximately 1.1–1.2 times wider than long, widest across eyes, slightly narrower than pronotum; eyes moderate in size and prominent, about 1.0–1.1 times longer than tempora; infraorbital carina incomplete; gular sutures moderately separated, diverged basally. Antennae dilated apically; antennomeres 1–3 elongate, 1 longest, 4–10 distinctly transverse, 11 longer than wide, slightly longer than preceding two combined. *Thorax.* Pronotum slightly transverse, approximately 1.2–1.3 times wider than long, widest at apical third; pubescence directed anteriorly in midline. Elytra slightly transverse, slightly wider than pronotum, elytron approximately 1.7 times longer than wide, pubescence directed posteriorly and postero-laterally; postero-lateral margin almost straight; hind wings fully developed. *Legs.* Slender and long, with dense pubescence and setae; tibiae with two spurs at apex; meso- and metatarsomeres 1–4 subequal in length. *Abdomen.* Parallel-sided, widest at middle; surface glossy and densely pubescent; male tergite VIII with 4 macrosetae on each side of midline, posterior margin broadly truncate, slightly emarginate; posterior margin of male sternite VIII rounded. *Genitalia.* Median lobe oval, apical process convergent at apex in ventral aspect. Apical lobe of paramerites elongate. Spermatheca with large bursa, duct long and slender, coiled apically.

##### Distribution.

Korea (North), Europe (Austria, Belgium, Bulgaria, Croatia, Czech Republic, Finland, France, Great Britain, Germany, Hungary, Italy, The Netherlands, Norway, Poland, Romania, Slovakia, Sweden and Switzerland) and Russia (Caucasus and East Siberia).

##### Remarks.

This species was recorded by Paśnik (2001) in North Korea and a dissected specimen was unavailable. Accordingly, we could not describe the mouthparts and aedeagus in detail. This species has been known to occur on disc fungi (Palm 1970).

#### Atheta (Microdota) pasniki

Taxon classificationAnimaliaColeopteraStaphylinidae

Lee & Ahn sp. n.

http://zoobank.org/C6A4FB75-A11F-4732-805E-E47039541DF3

Figs 10 , 22–27 , 33 , 42 , 51 , 60 , 69 , 78

##### Material examined.

Holotype, labeled as follows: ‘KOREA: Gangwon Prov., Pyeongchang-gun, Jinbu-myeon, Dongsan-ri, Mt. Odaesan, Sangwonsa, 4 VI 2001, S.-J. Park, *ex* sifting, HOLOTYPE Atheta (Microdota) pasniki Lee and Ahn, Desig. S.-G. Lee and K.-J. Ahn 2015’ Deposited in CNUIC, Daejeon. Paratypes, 40 exx. (total); 5 exx., same data as holotype; 8 exx., same data as holotype except ‘N37°47.074' E128°33.735', 15 V 2006, S.-J. Park, Y.-H. Kim, *ex* near stream’; 4 exx., same data as holotype except ‘N37°47.104' E128°33.57.2', 10 V 2007, TK Kim, sifting’; 2 exx., same data as holotype except ‘8 V 2004,S.J.Park, D.H. Lee, S. M. Choi *ex* sifting; 1 ex., same data as holotype except ‘25 - 26 IV 2001, K.-J. Ahn, *ex* sifting’; 2 exx., Gangwon Prov., Pyeongchang-gun, Mt. Odaesan, Sangwonsa, 26 IV 2001, M.-J. Jeon’; 1 ex., same data as former except ‘S.-J. Park, *ex* near stream; 8 exx., same data as the former except ‘25 V 2004, S J Park, J S Park, *ex* sifting; 1 ex., Gangwon Prov., Hongcheon-gun, Nae-myeon, Mt. Gyebangsan, Unduryeong, N37°42.49.9' E128°26.40.5', 1100m, 11 V 2007, TK Kim, *ex* leaf litter near stream; 5 exx., Kangwon Prov., Inje-gun, Yongdaeri, 13 IX 1998, K.-J. Ahn, K.-L. You, H.-J. Lim, *ex* leaf litter.

##### Description.

Length 1.7–2.3 mm. Body (Fig. 10) parallel-sided, more or less flattened dorso-ventrally; surface glossy, densely pubescent, with fine microsculpture. Body usually reddish brown; elytra paler than other parts; legs yellowish brown. *Head.* Subcircular, approximately 1.0–1.1 times wider than long, widest behind eyes, slightly narrower than pronotum; eyes small, about 0.8 times longer than tempora; gular sutures moderately separated, diverged basally; infraorbital carina incomplete; cervical carina complete. Antennae (Fig. 33) dilated apically; antennomeres 1–3 elongate, 1 longest, 4–10 transverse, 11 longer than wide, about as long as preceding two combined. *Mouthparts.* Labrum (Fig. 22) transverse, anterior margin emarginate; two lateral sensilla and about 8 macrosetae present on each side of midline; α-sensillum long and setaceous, more than twice longer than ε-sensillum, β- and γ-sensilla reduced; epipharynx as in Fig. 23. Mandibles asymmetrical, pointed apically, about 1.6–1.7 times as long as basal width; right one (Fig. 24) with small internal tooth, anterior margin serrulate; prostheca developed. Lacinia of maxilla with seven spines in distal comb, two isolated spines present; maxillary palpus elongate, with pubescence and long setae; palpomere 1 smallest and about 1.7–1.8 times as long as wide, 2 about 2.3–2.5 times longer than wide, 3 longer than 2, about 2.2–2.4 times as long as wide, 4 digitiform, filamentous sensilla not reaching to basal half (Fig. 25). Labium (Fig. 26) with ligula divided into two lobes in basal half; two medial setae contiguous; two basal pores closed together; lateral pseudopores, one setal pore and two real pores present on prementum; labial palpus elongate, with many setulae; palpomere 1 largest and dilated basally, about 1.4–1.6 times longer than wide, with γ-setula close f-setula, 2 shortest, about 1.5–1.7 times longer than wide, 3 about as long as 1, about 2.2–2.4 times longer than wide. Mentum (Fig. 27) trapezoidal, anterior margin emarginate, v-seta relatively long. *Thorax.* Pronotum transverse, approximately 1.3 times wider than long, widest at apical third; pubescence directed anteriorly. Metanotal scutum with one long seta and about two short setae on each side of midline; mesocoxal cavities narrowly separated, mesoventral process pointed at apex, about as long as isthmus and metaventral process combined. Elytra relatively short, transverse, slightly wider than pronotum; elytron approximately 1.6–1.7 times longer than wide, pubescence directed postero-laterally; postero-lateral margin almost straight; hind wings fully developed; flabellum composited one setose lobe. *Legs.* Slender and long, with dense pubescence and setae; tibia with two spurs at apex; length ratio of tarsomeres 19:20:22:57 (protarsus); 22:25:26:26:53 (mesotarsus); 31:33:33:33:68 (metatarsus); one empodial seta present, very short. *Abdomen.* Subparallel-sided, widest at middle; surface glossy and densely pubescent, with fine and imbricate microsculpture; macrochaetal arrangement of tergites II–VI 01-02-12-12-13; male tergite VIII (Fig. 42) with 4 macrosetae on each side of midline, posterior margin subtruncate; male sternite VIII with 7 macrosetae on each side of midline, posterior margin convex, subtriangular, with marginal setae; posterior margin of female tergite VIII truncate; posterior margin of female sternite VIII slightly emarginate at middle, with long and short marginal setae; minute setae present in median region. *Genitalia.* Median lobe (Figs 51, 60) oval; apical process parallel-sided, convergent at apex in ventral aspect. Apical lobe of paramerites (Fig. 69) elongate and parallel-sided with four setae, subequal in length. Spermatheca (Fig. 78) with large bursa and small umbilicus; duct relatively short, one coiled apically.

##### Distribution.

Korea (South).

##### Remarks.

This species is similar to Atheta (Microdota) nakanei, but can be distinguished by shape and structure of internal sac of aedeagus. They were usually collected near streams or in moist regions of forest of Gangwon province by sifting litter.

##### Etymology.

Named after Grzegorz Paśnik in honor of his research on Korean Athetini.

#### Atheta (Microdota) silvatica

Taxon classificationAnimaliaColeopteraStaphylinidae

Bernhauer, 1907

Fig. 11

Atheta (Microdota) silvatica Bernhauer, 1907: 405.
Atheta (Amidobia?) silvatica : Sawada 1974: 184 (as valid species).
Atheta (Microdota) silvatica : Paśnik 2001: 209; Smetana 2004: 388 (as valid species).

##### Material examined.

Syntype, 1♂, labeled as follows: ‘Japan. Sauter Negishi, 25. 2. 05, silvatica bernh typ. det Bernhauer, silvatica Bernh. Typus, Chicago NHMus M.Bernhauer Collection’. NORTH KOREA: 1 ex., Korea 12.6.1974 Vaudo ad Nampo Exp. Inst.Zool.Cr. [North Korea, Pyeongannam Prov., Nampo-si, Waudo, 12.vi.1974, ISEA].

##### Description.

Length about 1.5–2.0 mm. Body (Fig. 11) slender and parallel-sided, more or less flattened; surface distinctly glossy and densely pubescent, with fine microsculpture. Body usually reddish brown; head slightly darker than pronotum and elytra; legs yellowish brown. *Head.* Subquadrate, approximately 1.0–1.1 times wider than long, widest across eyes, slightly narrower than pronotum; eyes small, about 0.8 times longer than tempora; infraorbital carina complete; gular sutures moderately separated, diverged basally. Antennae dilated apically, longer than head and pronotum combined; antennomeres 1–3 elongate, 1 longest, 4 about as long as wide, 5–10 distinctly transverse, 11 longer than wide, about as long as preceding two combined. *Thorax.* Pronotum transverse, approximately 1.3–1.4 times wider than long, widest at apical third; pubescence directed anteriorly in midline. Elytra slightly transverse, slightly wider than pronotum, elytron approximately 1.7–1.8 times longer than wide, pubescence directed posteriorly and postero-laterally; postero-lateral margin almost straight; hind wings fully developed. *Legs.* Slender and long, with dense pubescence and setae; tibiae with two spurs at apex; meso- and metatarsomeres 1–4 subequal in length. *Abdomen.* Parallel-sided, widest at middle; surface distinctly glossy and densely pubescent; male tergite VIII with 4 macrosetae on each side of midline, posterior margin emarginated at middle; male sternite VIII with 8 macrosetae on each side of midline, posterior margin broadly rounded. *Genitalia.* Median lobe oval, apical process slightly decurved at apex in lateral aspect.

##### Distribution.

Korea (North) and Japan.

##### Remarks.

This species was recorded by Paśnik (2001) in North Korea and a dissected specimen was unavailable. Accordingly, we could not describe the mouthparts and aedeagus in detail. This species is distinguished from similar species by the characters provided in Sawada (1974).

#### Atheta (Microdota) sogamensis

Taxon classificationAnimaliaColeopteraStaphylinidae

Paśnik, 2001

Fig. 12

Atheta (Microdota) sogamensis Paśnik, 2001: 210; Smetana 2004: 388 (as valid species).

##### Material examined.

Holotype, ♂, labeled as follows: ‘KOREA-SOKAM distr-SUNAN lg. PAWLOWSKI VIII 1971; HOLOTYPE Atheta (Microdota) sogamensis sp. n. det. G. Paśnik 2000’ [North Korea, Pyeongannam Prov., Pyeongyang-si, Sogam, viii.1971, Pawlowski].

##### Description.

Length about 2.0 mm. Body (Fig. 12) slender and parallel-sided, more or less flattened; surface distinctly glossy and densely pubescent, with fine microsculpture. Body usually reddish brown to dark brown; head almost black; elytra paler than other parts; legs yellowish brown. *Head.* Slightly transverse, approximately 1.1–1.2 times wider than long, widest across eyes, slightly narrower than pronotum; eyes large and prominent, about 1.5 times longer than tempora; infraorbital carina incomplete; gular sutures moderately separated, diverged basally. Antennae dilated apically, longer than head and pronotum combined; antennomeres 1–3 elongate, 1 longest, 4 about as long as wide, 5–10 distinctly transverse, 11 longer than wide, about as long as preceding two combined. *Thorax.* Pronotum transverse, approximately 1.3–1.4 times wider than long, widest at apical third; pubescence directed anteriorly in midline. Elytra slightly transverse, slightly wider than pronotum, elytron approximately 1.7 times longer than wide, pubescence directed posteriorly and postero-laterally; postero-lateral margin almost straight; hind wings fully developed. *Legs.* Slender and long, with dense pubescence and setae; tibiae with two spurs at apex; meso- and metatarsomeres 1–4 subequal in length. *Abdomen.* Parallel-sided, widest at middle; surface distinctly glossy and densely pubescent; male tergite VIII with 4 macrosetae on each side of midline, posterior margin broadly rounded; male sternite VIII with 7 macrosetae on each side of midline, posterior margin rounded. *Genitalia.* Median lobe (Paśnik 2001: figs 28–29) elongated oval, apical process convergent at apex in ventral aspect.

##### Distribution.

Korea (North).

##### Remarks.

This species was recorded by Paśnik (2001) in North Korea and a dissected specimen was unavailable. Accordingly, we could not describe the mouthparts and aedeagus in detail.

#### Atheta (Microdota) spiniventris

Taxon classificationAnimaliaColeopteraStaphylinidae

Bernhauer, 1907

Figs 13 , 34 , 43 , 52 , 61 , 70 , 79

Atheta (Microdota) spiniventris Bernhauer, 1907: 402; Smetana 2004: 388 (as valid species).
Atheta (Microdota) spinicauda Bernhauer, 1907: 404 (as valid species); Sawada 1974: 149 (as synonym of *Atheta spiniventris*).
Atheta (Amidobia) spiniventris ; Sawada 1974: 149 (as valid species).

##### Material examined.

Syntype, 1 ex., labeled as in Fig. 86. SOUTH KOREA: Chungbuk Prov.: 109 exx., Danyang-gun, Yeongchun-myeon, Mt. Taehwasan, 14.vii–14.viii.2001, KJ Ahn, SJ Park, CW Shin, FIT; Chungnam Prov.: 11 exx., Gongju-si, Mt. Gyeryongsan, Nammaetap, N36°21'11.8", E127°13'20.8",, 1–18.vi.2004, SM Choi, SJ Park, FIT; 7 exx., same data as the former except ‘Eunseon waterfall, N36°20'58.7", E127°12'41.3",’; 6 exx., same data as the former except ‘Donghaksa, N36°21'17.4", E127°14'55.7",’; 8 exx., Gongju-si, Uidang-myeon, Yongam-ri, 25.vii.2000, MH Kim, mushroom; 10 exx., Daejeon-si, Yuseong-gu, Chungnam National University, 18.vi–15.vii.2003, JH Choi, DH Lee, SM Choi, FIT; 3 exx., same data as the former except ‘4–18.vi.’; Gangwon Prov.: 13 exx., Hoengseong-gun, Gangrim-myeon, Bugok-ri, Mt. Chiaksan, 15.viii.2000, MH Kim, mushroom; 136 exx., Pyeongchang-gun, Cheondong-ri, Mt. Sambangsan, 13.vii–15.viii.2001, KJ Ahn, SJ Park, CW Shin, FIT in *Pinus* forest; 34 exx., Sokcho-si, Mt. Seoraksan, Biseondae, 30.vii–15.ix.2002, SJ Park, JS Park, FIT; Gyeonggi Prov.: 30 exx., Yangju-gun, Jangheung-myeon, Songchu-ri, Mt. Bukhansan, 26.vii–31.viii.2001, KJ Ahn, SJ Park, CW Shin, FIT; Jeonnam Prov.: 13 exx., Jangseong-gun, Mt. Naejangsan, Baekyangsa, 15.vi.2000, US Hwang, HJ Kim, Dung.

##### Description.

Length 1.6–2.1 mm. Body (Fig. 13) relatively broad and subparallel-sided, surface slightly glossy and densely pubescent, with slightly coarse punctures. Body dark yellow to yellowish brown; head dark brown to black; basal articles of antennae paler; abdominal segments V–VII darker than other segments; legs yellowish brown. *Head.* Slightly transverse, approximately 1.1–1.2 times wider than long, widest across eyes, slightly narrower than pronotum; eyes distinctly large and prominent, about 1.8–2.0 times as long as tempora; infraorbital carina complete; gular sutures moderately separated; cervical carina complete. Antennae (Fig. 34) long and slender; antennomeres 1–3 elongate, 1 longest, 4–10 distinctly transverse, 11 about as long as 1, slightly longer than preceding two combined. *Mouthparts.* Labrum transverse, anterior margin slightly emarginate; two lateral sensilla and about 8 macrosetae present on each side of midline, α-sensillum setaceous, about twice as long as ε-sensillum, β- and γ-sensilla reduced. Mandibles asymmetrical, pointed apically, approximately 1.5–1.6 times longer than wide; right one with small internal tooth, anterior margin serrulate; prostheca developed. Lacinia of maxilla with seven spines in distal comb, two isolated spines present, distal comb and isolated spines close; maxillary palpus elongate, with pubescence and long setae; palpomere 1 smallest and about twice as long as wide, 2 about 2.4–2.7 times longer than wide, 3 slightly longer than 2, about 2.4–2.6 times as long as wide, 4 digitiform; filamentous sensilla reaching to basal half. Labium with ligula narrowly long, divided into two lobes in basal half; two medial setae narrowly separated; two basal pores closed together; median pseudopores absent; lateral pseudopores, one setal pore and two real pores present on prementum; palpus elongate, with many setulae; palpomere 1 largest and dilated basally, about 1.4–1.5 times longer than wide, γ-setula located between α- and b-setulae, closer b than α-setula, 2 shortest and about 1.3–1.5 times longer than wide, 3 more or less dilated apically, about as long as 1, about 3.0 times longer than wide. *Thorax.* Pronotum transverse, approximately 1.3–1.4 times wider than long, widest in apical third; pubescence directed anteriorly in midline. Metanotal scutum with one long seta and two short setae on each side of midline; mesocoxal cavities narrowly separated, mesoventral process more or less pointed at apex, as long as or slightly longer than isthmus and metaventral process combined; isthmus as long as or shorter than metaventral process. Elytra slightly wider than pronotum; elytron approximately 1.5–1.6 times longer than wide; pubescence directed posteriorly and postero-laterally; postero-lateral margin almost straight; hind wings fully developed, flabellum composited 4 setose lobes. *Legs.* Slender and long, with dense pubescence and setae; tibia with two spurs at apex; length ratio of tarsomeres 14:16:18:50 (protarsus); 18:20:22:21:51 (mesotarsus); 25:25:25:25:59 (metatarsus); one empodial seta present, shorter than claw. *Abdomen.* Parallel-sided, widest at middle; surface glossy and densely pubescent, with fine and imbricate microsculpture; macrochaetal arrangement of tergites II–VI 01-02-12-12-13; male tergite VIII (Fig. 43) with 4 macrosetae on each side of midline, posterior margin with two outer process and about 4–5 inner process, outer process longer than inner process decurved, slightly pointed at apex, inner process variable and blunt at apex, shorter than outer process; male sternites IV–VI with many pores in anterior margin, VIII with 7 macrosetae on each side of midline, posterior margin broadly rounded, with long marginal setae; posterior margin of female sternite VIII truncate, with long and short marginal setae. *Genitalia.* Median lobe (Figs 52, 61) oval; apical process elongate and convergent at apex in ventral aspect; internal sac complicated. Apical lobe of paramerites (Fig. 70) very long with short four setae; c- and d-setae longer than a- and b-setae, subequal in length, positioned apically. Spermatheca (Fig. 79) with small umbilicus, duct short and compactly coiled.

##### Distribution.

Korea (South) and Japan.

##### Remarks.

This species is very similar to Atheta (Microdota) vagans, but can be distinguished by the minute characters provided in Sawada (1974). Many specimens were found on mushrooms in forested habitats.

#### Atheta (Microdota) spinula

Taxon classificationAnimaliaColeopteraStaphylinidae

(Sawada, 1970)

Figs 14 , 35 , 44 , 53 , 62 , 71 , 80

Ischnopoda (Hygroecia) spinula Sawada, 1970: 60
Atheta (Amidobia) spinula : Sawada 1974: 175 (as valid species).
Atheta (Microdota) spinula : Smetana 2004: 388 (as valid species).

##### Material examined.

SOUTH KOREA: Chungnam Prov.: 20 exx., Gongju-si, Mt. Gyeryongsan, N36°20'27.8", E127°15'11.5",, 1–18.vi.2004, KJ Ahn, SM Choi, JS Park, FIT.

##### Description.

Length 1.4–1.8 mm. Body (Fig. 14) parallel-sided, more or less flattened dorso-ventrally; surface fairly glossy, densely pubescent, with fine microsculpture. Body usually yellowish brown; head and abdominal segments V–VII darker than other parts. *Head.* Subquadrate, about as wide as long, widest across eyes, slightly narrower than pronotum; eyes moderate in size and prominent, about 1.2–1.3 times longer than tempora; gular sutures moderately separated, diverged basally; infraorbital carina incomplete; cervical carina complete. Antennae (Fig. 35) dilated apically; antennomeres 1–3 elongate, 1 longest, 4–10 slightly transverse to transverse, 11 longer than wide, about as long as preceding two combined. *Mouthparts.* Labrum transverse, anterior margin emarginate; two lateral sensilla, about 8 macrosetae present on each side of midline; α-sensillum setaceous, about twice longer than ε-sensillum, β- and γ-sensilla reduced, convergent at apex. Mandibles asymmetrical, pointed apically, about 1.6–1.7 times as long as basal width; right one with small internal tooth, anterior margin serrulate; prostheca developed. Lacinia of maxilla with seven spines in distal comb, two isolated spines present; maxillary palpus elongate, with pubescence and long setae; palpomere 1 smallest and about 1.6–1.8 times as long as wide, 2 about 2.5–2.7 times longer than wide, 3 longer than 2, about 2.2–2.4 times as long as wide, 4 digitiform, filamentous sensilla reaching to basal half. Labium with ligula narrowly long, divided into two lobes in basal half; two medial setae narrowly separated, two basal pores closed together; lateral pseudopores, one setal pore and two real pores present on prementum; labial palpus elongate, with many setulae; palpomere 1 largest, about 1.3–1.5 times longer than wide, with γ-setula close b-setula, 2 shortest, about 1.2–1.4 times longer than wide, 3 about as long as 1, about 3.0 times longer than wide. *Thorax.* Pronotum transverse, approximately 1.3 times wider than long, widest at apical third; pubescence directed anteriorly in midline. Metanotal scutum with one long seta and three short setae on each side of midline; mesocoxal cavities narrowly separated, mesoventral process pointed at apex, longer than metaventral process; length ratio of mesoventral process, isthmus and metaventral process 6:4:3. Elytra slightly wider than pronotum; elytron approximately 1.7 times longer than wide, pubescence directed postero-laterally; postero-lateral margin almost straight; hind wings fully developed; flabellum composited one setose lobe. *Legs.* Slender and long, with dense pubescence and setae; tibia with two spurs at apex; length ratio of tarsomeres 16:17:19:49 (protarsus); 20:22:23:23:48 (mesotarsus); 28:28:28:27:60 (metatarsus); one empodial seta present, shorter than claw. *Abdomen.* Parallel-sided, widest at middle; surface distinctly glossy and densely pubescent, with fine and imbricate microsculpture; macrochaetal arrangement of tergites II–VI 01-02-12-12-13; male tergite VIII (Fig. 44) with 4 macrosetae on each side of midline, posterior margin slightly emarginate; male sternites V–VII with many pores in anterior margin, VIII with 7 macrosetae on each side of midline, posterior margin slightly rounded, with marginal setae; posterior margin of female tergite VIII similar to male; posterior margin of female sternite VIII slightly emarginate at middle, with long and short marginal setae; minute setae present in median region. *Genitalia.* Median lobe (Figs 53, 62) oval, apical process more or less narrow apically, blunt at apex in ventral aspect. Apical lobe of paramerites (Fig. 71) subtriangular, narrow apically with four setae; a-seta longest, b-seta longer than c- and d- setae. Spermatheca (Fig. 80) with round bursa, duct relatively long, coiled apically.

##### Distribution.

Korea (South) and Japan.

##### Remarks.

This species has been known to be collected near marsh (Sawada 1970). All Korean specimens were collected by FIT in forest.

#### Atheta (Microdota) subcrenulata

Taxon classificationAnimaliaColeopteraStaphylinidae

Bernhauer, 1907

Figs 15 , 36 , 45 , 54 , 63 , 72 , 81

Atheta (Microdota) subcrenulata Bernhauer, 1907: 403; Paśnik 2001: 209; Smetana 2004: 388 (as valid species).
Atheta (Amidobia) subcrenulata : Sawada 1974: 166 (as valid species).

##### Material examined.

Syntype, 2♂♂, labeled as in Figs 87–88. NORTH KOREA: 1 ex., Korea 21–25. 5. 74 pr. Ćhongdźin-si Exp. Inst.Zool Cr. [North Korea, Hamgyeongbuk Prov., Cheongjin-si, 21–25.v.1974, ISEA]; 1 ex., Korea 1981 Pekson-ri A. Szeptycki [North Korea, Hwanghae Prov., Mt. Suyangsan, 15.vi.1981, A. Szeptycki]. SOUTH KOREA: Chungnam Prov.: 12 exx., Nonsan-si, Beolgok-myeon, N36°09'10.5", E127°18'24.9", 236 m, 22.v.2011, IS Yoo, decaying red pepper; 2 exx., Daejeon-si, Seo-gu, Jangan-dong, Jangtaesan Recreational Forest, N36°13'4.32", E127°20'34.44", 257 m, 17.iii.2011, IS Yoo, YH Kim, SG Lee, leaf litters; Chungbuk Prov.: 6 exx., Yeongdong-gun, Sangchon-myeon, Mulhan-ri, Mt. Minjujisan, N36°03’35.2", E127°52’31.3",, 518 m, 18.v.2011, JG Lee, TK Kim, decaying persimmon; Gangwon Prov.: 11 exx., Inje-gun, Inje-eup, Deoksan-ri, N38°04'46.1", E128°14'08.0", 384 m, 11.vi.2011, YH Kim, JH Song, SG Lee, decaying vegetables; Jeju Prov.: 10 exx., Jeju-si, Aewol-eup, N33°22'29.3", E126°30'37.9",, 21.v.2006, SI Lee, decaying vegetables.

##### Description.

Length about 1.5–2.0 mm. Body (Fig. 15) slender and parallel-sided, more or less flattened dorso-ventrally; surface fairly glossy, densely pubescent, with fine microsculpture. Body usually reddish brown to dark brown; head and abdomen almost black, darker than other parts; legs yellowish brown. *Head.* Subquadrate, approximately 1.0–1.1 times wider than long, widest across eyes, slightly narrower than pronotum; eyes moderate in size and prominent, about 1.0–1.2 longer than tempora; gular sutures moderately separated, diverged basally; infraorbital carina complete; cervical carina complete. Antennae (Fig. 36) dilated apically; antennomeres 1–3 elongate, 1 longest, 4–10 transverse, 11 about as long as preceding two combined. *Mouthparts.* Labrum transverse, anterior margin emarginate; two lateral sensilla and about 8 macrosetae present on each side of midline; α-sensillum long and setaceous, twice longer than ε-sensillum, β- and γ-sensilla reduced. Mandibles asymmetrical, pointed apically, approximately 1.6–1.7 times as long as basal width; right one with small internal tooth, anterior margin serrulate; prostheca developed. Lacinia of maxilla with seven spines in distal comb region, two isolated spines present; maxillary palpus elongate, with pubescence and long setae; palpomere 1 smallest and about twice as long as wide, 2 about 2.5–2.6 times longer than wide, 3 slightly longer than 2, about 2.5–2.7 times as long as wide, 4 digitiform, filamentous sensilla convergent apically, reaching to basal half. Labium with ligula divided into two lobes in basal half; two medial setae closed together; two basal pores narrowly separated; lateral pseudopores, one setal pore and two real pore present on prementum; labial palpus elongate, with many setulae; palpomere 1 largest, about 1.4–1.5 times longer than wide, with γ-setula close b-setula, 2 shortest, about 1.2–1.4 times longer than wide, 3 dilated apically, about 2.3–2.5 times longer than wide. *Thorax.* Pronotum transverse, approximately 1.3 times wider than long, widest at apical third; midline of pubescence directed anteriorly. Metanotal scutum with one long seta and two short setae on each side of midline; mesocoxal cavities narrowly separated, mesoventral process distinctly pointed at apex, longer than isthmus and metaventral process combined; metaventral process shorter than isthmus. Elytra slightly wider than pronotum; elytron approximately 1.6 times longer than wide, pubescence directed postero-laterally; postero-lateral margin almost straight; hind wings fully developed; flabellum composited five setose lobes. *Legs.* Slender and long, with dense pubescence and setae; tibia with two spurs at apex; length ratio of tarsomeres 18:19:20:47 (protarsus); 22:23:23:24:42 (mesotarsus); 30:29:29:26:50 (metatarsus); one empodial seta present, shorter than claw. *Abdomen.* Parallel-sided, widest at middle; surface fairly glossy and densely pubescent, with reticulate microsculpture; macrochaetal arrangement of tergites II–VI 01-02-12-12-13; male tergite VIII (Fig. 45) with 4 macrosetae on each side of midline, posterior margin truncate, minutely crenate; male sternites V–VII with many pores in anterior margin, VIII with 7 macrosetae on each side of midline, posterior margin slightly rounded, with long marginal seta; posterior margin of female tergite VIII subtruncate; posterior margin of female sternite VIII broadly rounded, with long and short marginal setae; minute setae present in median region. *Genitalia.* Median lobe (Figs 54, 63) oval, apical process convergent at apex in ventral aspect; internal sac complicated. Apical lobe of paramerites (Fig. 72) with four setae, subequal in length. Spermatheca (Fig. 81) with conical umbilicus, duct sinuate and coiled apically.

##### Distribution.

Korea (South, North), China (Beijing and Zhejiang) and Japan.

##### Remarks.

This species is very similar to Atheta (Microdota) amicula, but can be distinguished by the internal sac of median lobe and spermatheca.

### Species removed from the Korean fauna

#### Atheta (Microdota) scrobicollis

Taxon classificationAnimaliaColeopteraStaphylinidae

(Kraatz, 1859)

##### Material examined.

Syntype, 1 ex., labeled as follows: type, C. cava, Coll. Jil. Moser, Type. NORTH KOREA: 1 ex., Korea 81/51 Kwail 18.VI. A.S. [North Korea, Hwanghae Prov., Kwail, 18.vi.1981, A. Szeptycki].

##### Remarks.

The North Korean record of the species by Paśnik (2001) is a misidentification of other Atheta (Microdota) species. This species is unlikely to be found in Korea as its distribution has shown that it occurs in southern Europe and subtropical regions. This species differs from a syntype of Atheta (Microdota) scrobicollis by several diagnostic characters although we were not able to identify it due to a paucity of specimen.

#### Atheta (Microdota) kobensis

Taxon classificationAnimaliaColeopteraStaphylinidae

Cameron, 1933

##### Material examined.

Syntype, 3 exx., labeled as in Figs 89–91. NORTH KOREA: 2 exx., Korea 1981 Kymgangsan A. Szeptycki [North Korea, Gangwon Prov., Mt. Geumgangsan, 1.viii.1981, A. Szeptycki]; 1 ex., Korea 1981 Pekson-ri A. Szeptycki [North Korea, Hwanghae Prov., Baeksong-ri, 1981, A. Szeptycki].

##### Remarks.

The North Korean record of the species by Paśnik (2001) is a misidentification of other Atheta (Microdota) species. This species differs from the syntypes of Atheta (Microdota) kobensis by the shape of aedeagus and spermatheca. We cannot identify the species at this point because specimens are not in good shape.

## Discussion

*Microdota*, one of the most species-rich subgenus of *Atheta*, is taxonomically reviewed and the subgenus is represented in Korea by 15 species. Most specimens were collected by flight intercept trap and some others collected by sifting leaf litter. Six species [Atheta (Microdota) amicula, Atheta (Microdota) formicetorum, Atheta (Microdota) koreana, Atheta (Microdota) muris, Atheta (Microdota) palleola and Atheta (Microdota) spiniventris] have been found in association with fungus but they seem not to be mycetophagous. Atheta (Microdota) subcrenulata was usually found in decaying vegetables and straw piles. No habitat preference of North Korean species is available.

Since the first record of Korean *Microdota* species by Bernhauer (1923), 11 species were recorded in North Korea (Paśnik 2001). Two species are removed from the Korean fauna and six species are added to the Korean fauna in this study. Consequently, the number of *Microdota* species known to occur in the Korean Peninsula increases from 11 to 15. Five species [Atheta (Microdota) hamgyongsani, Atheta (Microdota) Jangtaesanensis Lee & Ahn, sp. n., Atheta (Microdota) kangsonica, Atheta (Microdota) pasniki Lee & Ahn, sp. n. and Atheta (Microdota) sogamensis] are found only in the Korean peninsula. Of the remaining species, two [Atheta (Microdota) formicetorum and Atheta (Microdota) silvatica] were previously known to occur in Japan and Korea, four [Atheta (Microdota) kawachiensis, Atheta (Microdota) muris, Atheta (Microdota) spiniventris, Atheta (Microdota) spinula] were previously considered endemic to Japan, two [Atheta (Microdota) koreana and Atheta (Microdota) subcrenulata] inhabit Korea, China and Japan, one [Atheta (Microdota) palleola] occurs in Korea, Europe and Russia and the last [Atheta (Microdota) amicula] is found in trans-Palaearctic, Nearctic and Neotropical regions.

Compared with other north temperate regions, such as Czech Republic (17 species), Great Britain (16 species), Spain (13 species), and Turkey (11 species), the diversity of Korean *Microdota* is close to that of the regions (Smetana 2004). However, it appears a bit lower than that of adjacent Japan (20 species). Although the Japanese archipelago is an area of relatively high endemism for *Microdota*, this study decreases the number of species considered endemic to this area from 14 (70%) to 11 (55%) (Smetana 2004). Without doubt, further collecting efforts and the study of Korean species will increase opportunity to discover additional Atheta (Microdota) species.

## Supplementary Material

XML Treatment for Microdota

XML Treatment for Atheta (Microdota) amicula

XML Treatment for Atheta (Microdota) formicetorum

XML Treatment for Atheta (Microdota) hamgyongsani

XML Treatment for Atheta (Microdota) jangtaesanensis

XML Treatment for Atheta (Microdota) kangsonica

XML Treatment for Atheta (Microdota) kawachiensis

XML Treatment for Atheta (Microdota) koreana

XML Treatment for Atheta (Microdota) muris

XML Treatment for Atheta (Microdota) palleola

XML Treatment for Atheta (Microdota) pasniki

XML Treatment for Atheta (Microdota) silvatica

XML Treatment for Atheta (Microdota) sogamensis

XML Treatment for Atheta (Microdota) spiniventris

XML Treatment for Atheta (Microdota) spinula

XML Treatment for Atheta (Microdota) subcrenulata

XML Treatment for Atheta (Microdota) scrobicollis

XML Treatment for Atheta (Microdota) kobensis
